# Supplementary material for: Allosteric coupling between α-rings of the 20S proteasome
Source: Nat Commun. 2020 Sep 11;11:4580. doi: 10.1038/s41467-020-18415-7 (PMC7486400; doi:10.1038/s41467-020-18415-7)
Supplement: Supplementary file 1 — Supplementary Information [file 41467_2020_18415_MOESM1_ESM.docx]

**Allosteric coupling between α-rings of the 20S proteasome**

Zanlin Yu^1^, Yadong Yu^1,†^, Feng Wang^1^, Alexander G. Myasnikov^1,‡^,

Philip Coffino^2^* and Yifan Cheng^1,3^*

^1^Department of Biochemistry and Biophysics, University of California San Francisco, CA, 94158

^2^Laboratory of Cellular Biophysics, Rockefeller University, New York, NY, 10065

^3^Howard Hughes Medical Institute, University of California San Francisco, San Francisco, CA 94158

*Correspondence

Philip Coffino ([pcoffino@mail.rockefeller.edu](mailto:pcoffino@mail.rockefeller.edu))

Yifan Cheng ([Yifan.cheng@ucsf.edu](mailto:Yifan.cheng@ucsf.edu))

^†^Current address: Immutics Inc., 1140A O’Brien, Menlo Park, CA 94025

^‡^Current address: Department of Structural Biology, St. Jude Children’s Research Hospital, 262 Danny Thomas Place, Memphis, TN 38105

**Supplementary Figures and Legends**

**Supplementary Tables and Legends**

**
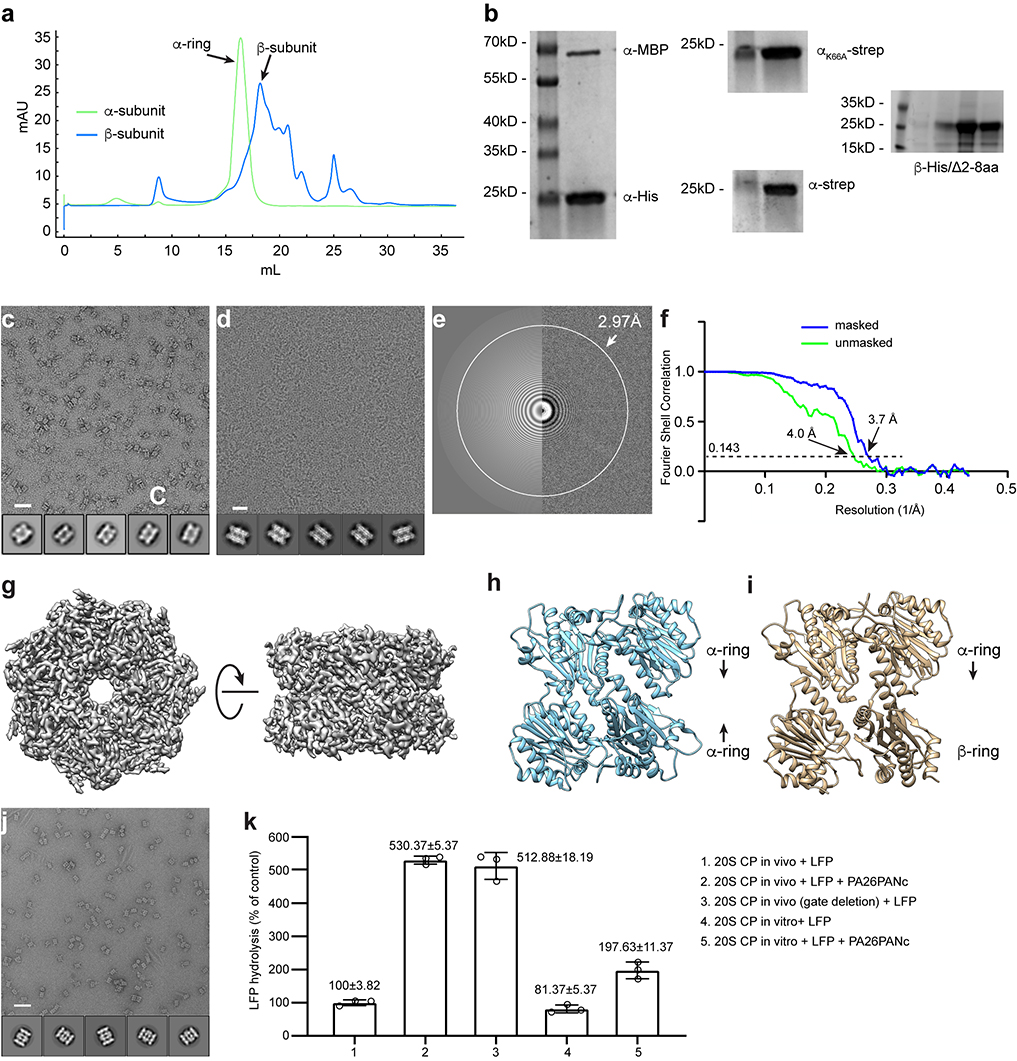
Supplementary Figure 1** *Expression and purification of individual subunits of archaeal 20S proteasome, and in vitro assembly of 20S proteasome CP.*

**a**: Size exclusion chromatography profiles of purified α-subunit as heptameric ring (green) and individual β-subunits (blue). **b**: SDS PAGE of purified α- and β-subunits with various affinity tags. For each protein purification, the SDS PAGE gels were used to examine the purity of the protein preparations, and only those showing adequate purity were used in the next step. **c**: A typical electron micrograph (scale bar 25 nm) and representative 2D class averages of negatively stained double α-rings. Purified α-subunits assemble into homo-heptameric rings, and two α-rings come together, forming a tetradecamer ring. **d**: A typical cryo-electron micrograph (scale bar 20 nm) of frozen hydrated double α-rings. Inserted are representative 2D class averages. **e**: Fourier power spectrum (right) and simulated Thon ring (left) calculated from the micrograph shown in **D**. The white ring indicates 2.97 Å. **f**: Fourier Shell Correlation of the 3D reconstruction of double α-rings. **g**: Top and side view of a 3D reconstruction of tetradecamer ring formed by two heptameric α-rings of archaeal proteasome packed back-to-back at a resolution of 3.7 Å. **h**: Ribbon diagram of α-subunits from the tetradecamer α-ring. **i**: Ribbon diagram of two α-subunits and two β-subunits taken from the fully assembled archaeal 20S proteasome. Note that the α-subunits in the paired α tetradecamer ring are interdigitated (**h**), similar to the interdigitation between α- and β−rings (**i**). **j**: A typical electron micrograph (scale bar 50 nm) and representative 2D class average (insert) of negative stained 20S proteasome assembled *in vitro*. They are indistinguishable from the recombinant archaeal 20S proteasome expressed in and purified from *E. coli*. **k**: Comparison of peptidase activity of recombinant 20S CP assembled *in vivo* (bars 1, 2, 3) and recombinant 20S CP assembled *in vitro* (bars 4, 5), and their activation by PA26PANc. Bars 1 and 3 report, respectively, the activity of intact *in vivo* 20S and *in vivo* 20S CP, but with the gate deletion mutation. All experiments contain 16 nM 20S CP and 10 μM LFP with or without 160nM PA26PANc. Peptidase activity is expressed as the LFP hydrolysis rate normalized to that of the control, *in vivo* 20S CP without activator. Three (n=3) biologically independent experiments were carried out to derive the mean and standard deviation. The means are represented by data bars and the standard deviations are represented as error bars.

**
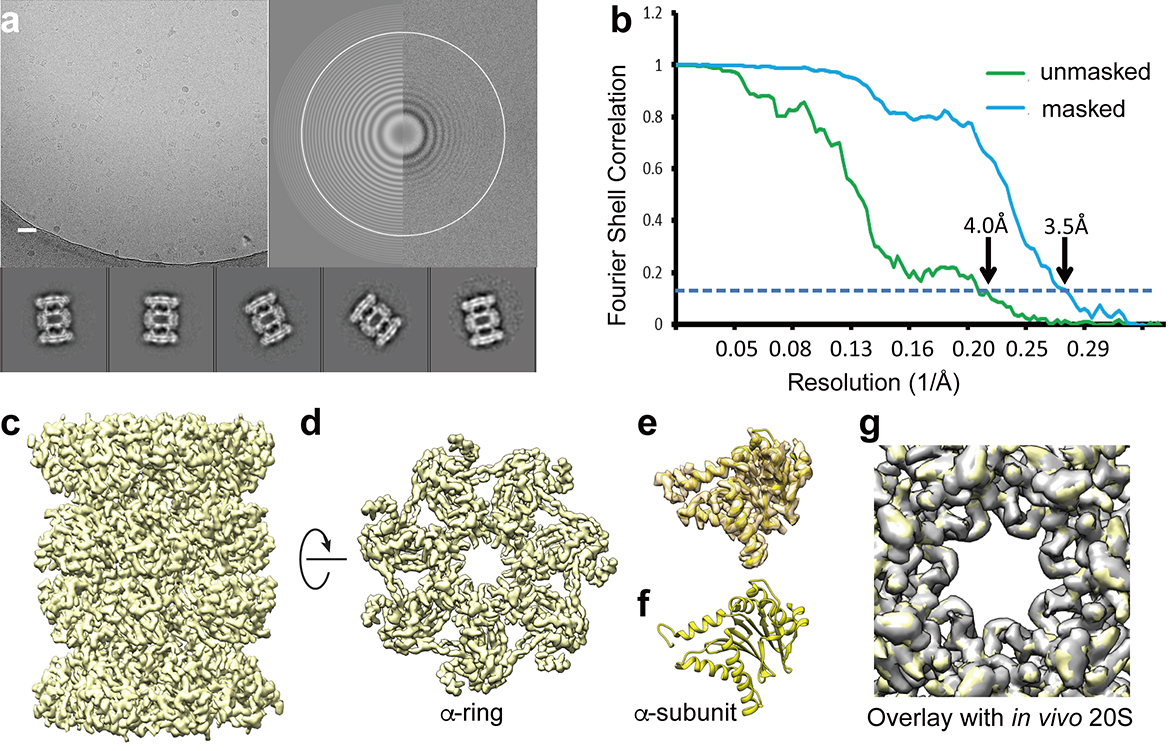
**

**Supplementary Figure 2**: *Single particle* *cryo-EM structure of* *in vitro assembled symmetric 20S CP* α_WT_ββα_WT_

**a**: A typical electron micrograph (scale bar 40 nm) of frozen hydrated α_WT_ββα_WT_ complex without substrate (upper left), Fourier power spectrum (ring indicates 3.77Å) calculated from the micrograph matched with simulated Thon rings (upper right), and typical 2D class averages of the complex (below). **b**: FSC curves between two half maps without (green) and with (blue) mask. Resolutions estimated by FSC=0.143 criterion is marked. **c**: Side view of the cryo-EM density map at a resolution of 3.5 Å*.* **d**: The top-view of α-ring density. **e**: Density of a single α-subunit (left) with atomic model docked. **f**: The atomic model of α-subunit of T20S showing the gate conformation its N-terminus. **g**: Overlay of the α-ring densities with that of 20S CP produced *in vivo* (EMD-5623).

**
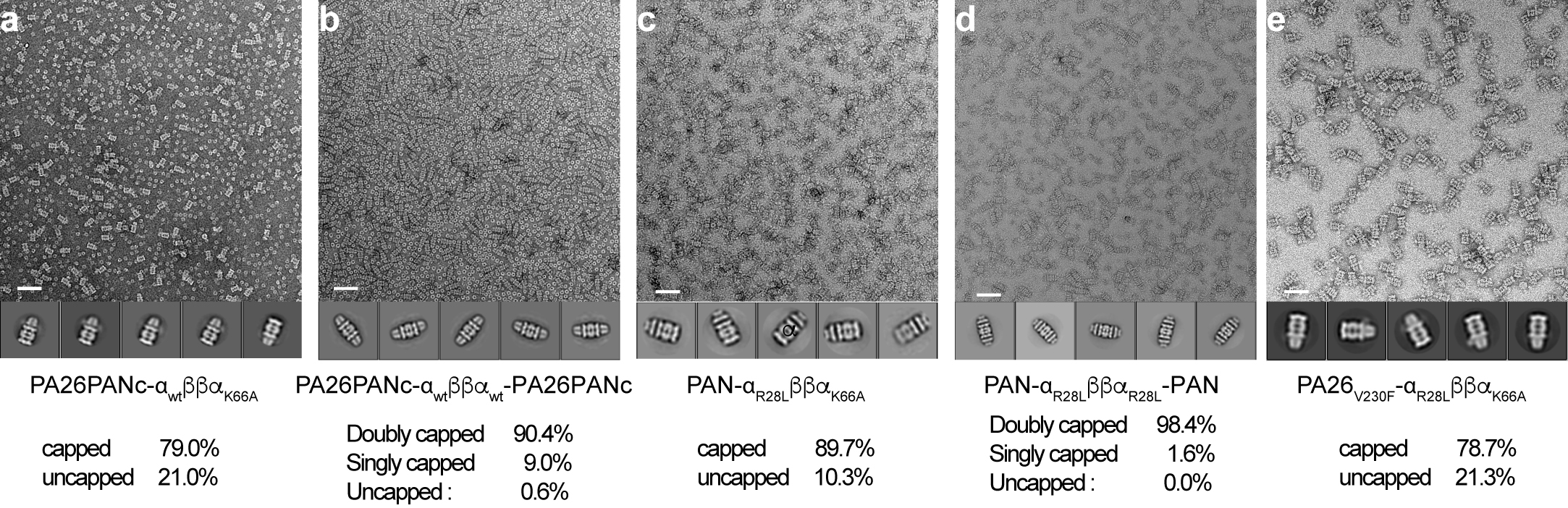
Supplementary Figure 3**: *In vitro assembly of symmetric and asymmetric proteasomes*

Representative negative stain EM images (scale bar 50 nm) and 2D class averages of asymmetric PA26PANc-α_WT_ββα_K66A_ complex (**a**), symmetric PA26PANc-α_WT_ββα_WT_-PA26PANc complex (**b**), asymmetric PAN-α_WT_ββα_K66A_ complex (with 1 mM ATP) (**c**), symmetric PAN-α_WT_ββα_WT_-PAN complex (with 1 mM ATP) (**d**) and asymmetric PA26V230F-α_WT_ββα_K66A_ complex (**e**). In all experiments, 16nM 20S CP and 160nM activator were incubated, and diluted (1:2~1:10) prior to preparing negative stain EM grids to obtain proper density of particles in grid. 2D class averages are obtained from particles autopicked from over 3 micrographs. Numbers of T20S CP capped with single, double or none activators are counted from one micrographs of each sample.

**
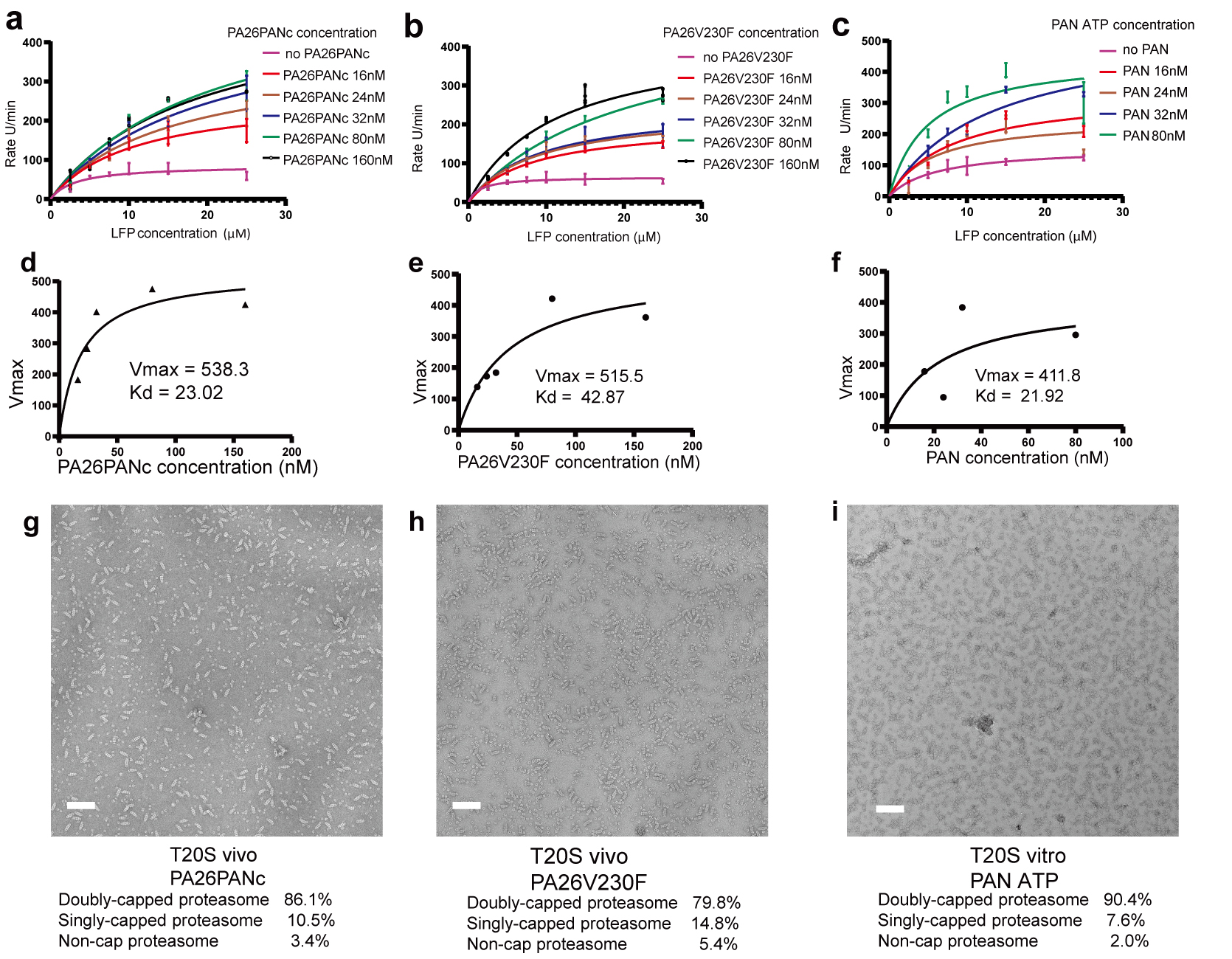
Supplementary Figure 4**: *Apparent binding affinity estimation of various activators of T20S CP*

**a** and **b**: Kinetics of LFP degradation of *in vivo* produced T20S incubated with indicated concentrations (0 to 160 nM) of PA26PANc (a), PA26(V230F) (b). **c**: Kinetics of LFP degradation of *in vitro* produced T20S(R82L) with indicated concentrations (0 to 80 nM) of PAN and 1 mM ATP (c). For each data point, three (n=3) biologically independent experiments were carried out. The means are used to derive the curve, and the error bar at each data point represents the standard deviation. **d** – **f**: For each experimental condition, the Vmax value at 0 nM cap is subtracted from Vmax table values to determine cap-stimulated Vmax values, and these are plotted against the concentration of activators used in a – c. Functional dependence of cap-stimulated Vmax on activator concentration is estimated by fitting to a hyperbolic curve and the apparent dissociation constant K_d-app_ then estimated as activator concentration producing half-maximum activation. **g** - **i**: Negative stain EM micrograph (scale bar 100 nm) of 16 nM *in vivo* produced T20S incubated with activator 112 nM PA26PANc (m) and 112 nM PA26V230F (n). The percent of doubly capped, singly capped and none-cap complexes are counted from the micrographs and listed below the micrograph. Negative stain EM micrograph of *in vitro* produced T20S (16 nM) incubated with activator 80 nM PAN with 1 mM ATP (o) are similarly presented. Numbers of T20S CP capped with single, double or none activators are counted from one micrographs of each sample.

**
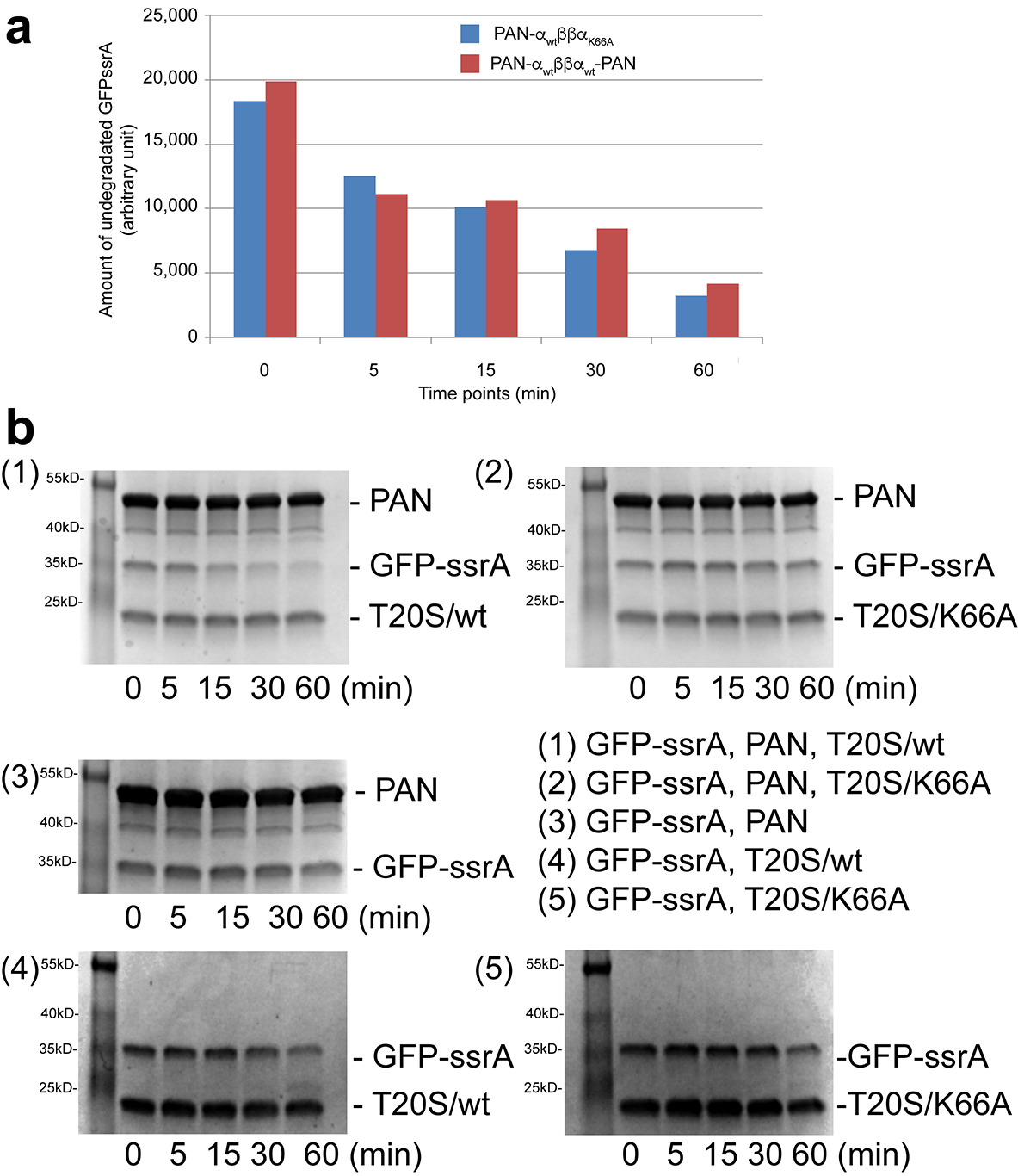
**

**Supplementary Figure 5:** *Degradation of GFPssrA by 20S-PAN complex*

**a**: Quantitative representation of the time course of GFP-ssrA degradation by PAN-α_WT_ββα_K66A_ and PAN-α_WT_ββα_WT_-PAN, determined by Coomassie Blue stained SDS PAGE, Figure 2c. Digitized GFPssrA band intensities are normalized to PAN band intensities (as loading control) and time-dependent GFPssrA degradation expressed in arbitrary units. **b**: Various control experiments demonstrating that GFP-ssrA degradation requires presence of an intact PAN-20S complex. Control conditions precluding PAN-20S assembly are T20S mutation K66A, which prevents PAN-20S association, omission of PAN, omission of 20S.

**
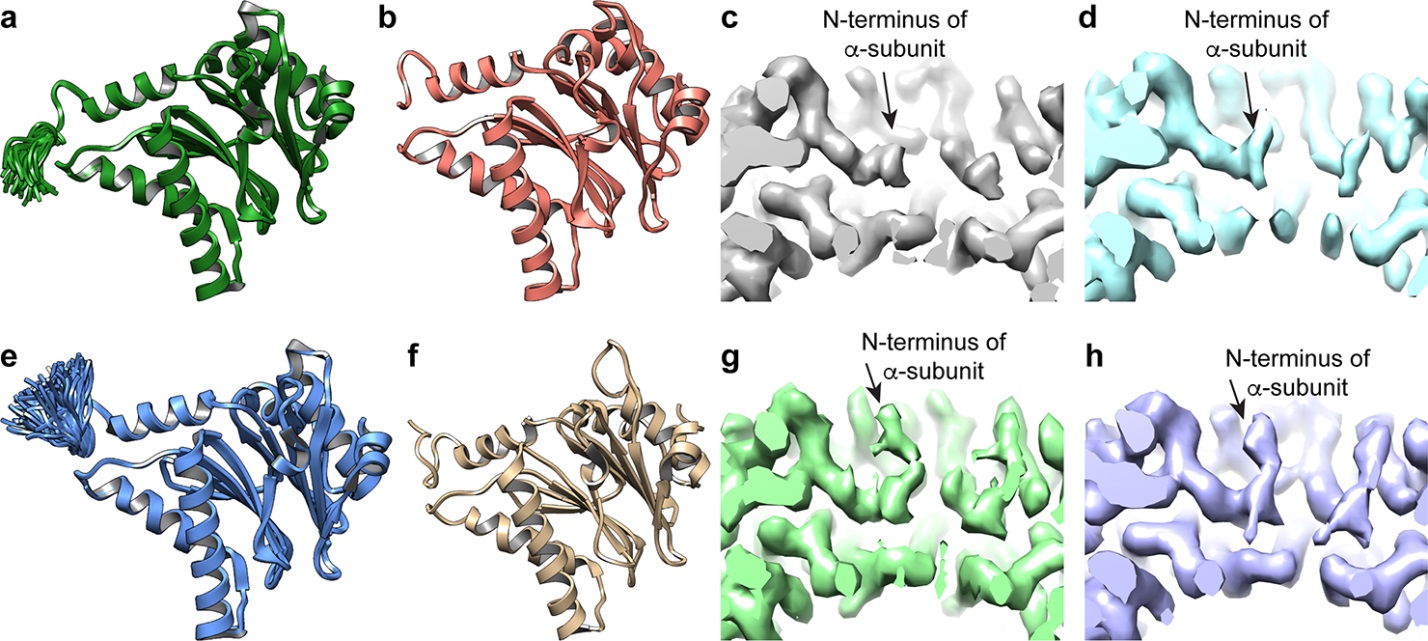
**

**Supplementary Figure 6**. *Comparison of the archaeal 20S proteasome in closed and open conformation*.

**a**: Ribbon diagram of NMR structures of α-subunit from archaea 20S CP in closed conformation ^1^, in which the N-termini of α-subunits point upwards. **b**: Ribbon diagram of α-subunit taken from single particle cryo-EM reconstruction of T20S CP without activator ^2^, where the gate is in closed conformation (by definition). **c**: Cryo-EM density of T20S CP in the gate region of α-subunit, viewed from middle of the central pore. d: The same views of cryo-EM density of the gate region within the distal α-subunit of PA26V230F-α_WT_ββα_K66A_ complex. The location of N-terminus of a α-subunit in both c and d is pointed by an arrow. **e**: Ribbon diagram of NMR structures of α-subunit in closed conformation ^1^, in which the N-termini of α-subunits point downwards. **f**: Ribbon diagram of α-subunit taken from the crystal structure of PA26PANc-20S complex ^3^, where the gate in α-ring is open. **g**: The same views of cryo-EM density of the gate region within the distal α-subunit of PA26PANc-α_WT_ββα_K66A_ complex. The location of N-terminus of a α-subunit in both c and d is pointed by an arrow. **h**: The same views of cryo-EM density of the gate region within the distal α-subunit of PAN-α_WT_ββα_K66A_ complex. The location of N-terminus of a α-subunit in both g and h is pointed by an arrow.

**
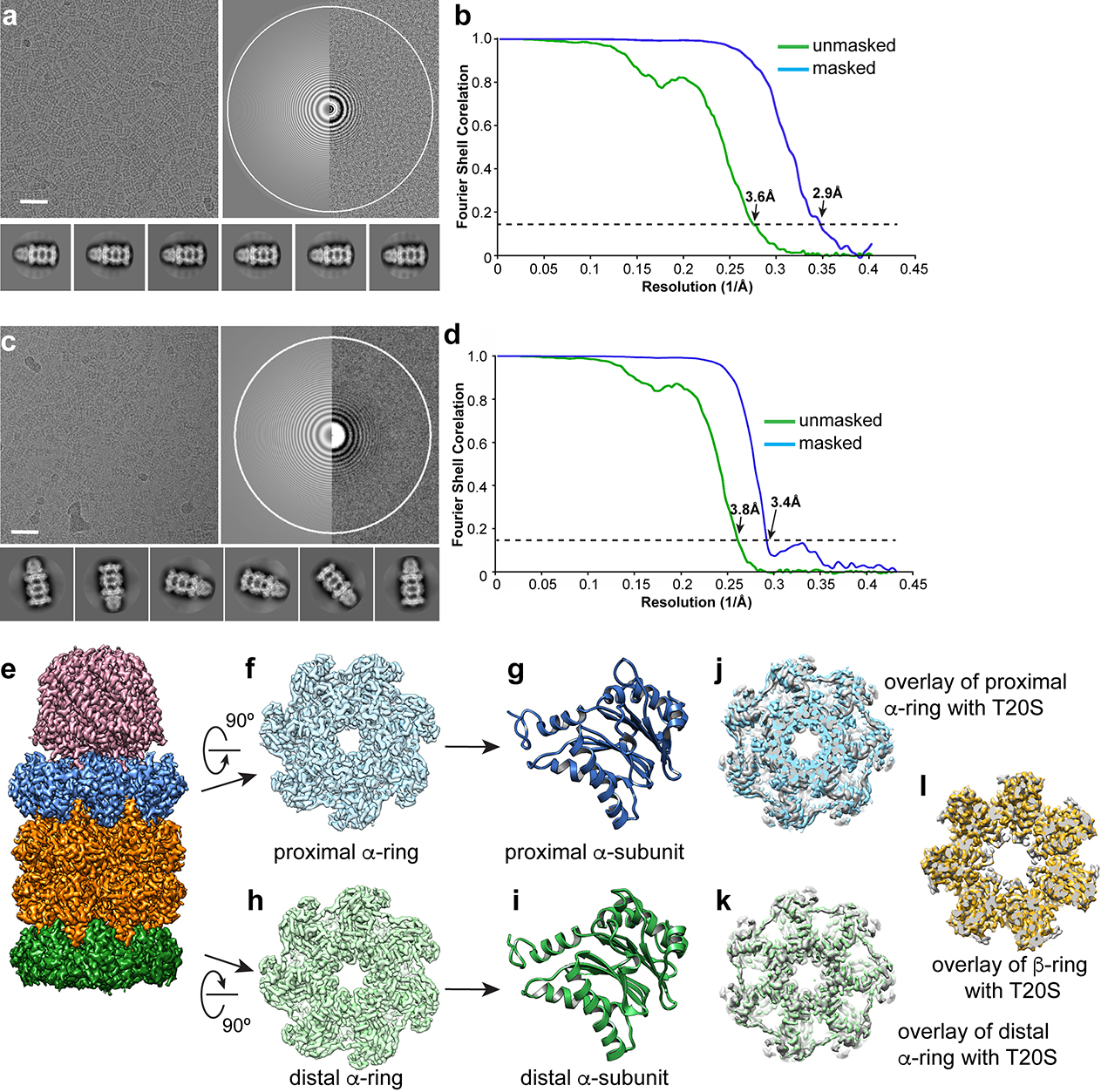
Supplementary Figure 7**. *Single particle cryo-EM of asymmetric* PA26PANc-α_WT_ββα_K66A_ *complex without and with peptide substrate LFP*

**a**: A typical electron micrograph (scale bar 40 nm) of frozen hydrated PA26PANc-α_WT_ββα_K66A_ complex without substrate (left), Fourier power spectrum calculated from the micrograph matched with simulated Thon rings (right), and typical 2D class averages of the complex. **b**: FSC curves between two half maps without (green) and with (blue) mask. Resolutions estimated by FSC=0.143 criterion is marked. **c**: A typical electron micrograph (scale bar 40 nm) of frozen hydrated PA26PANc-α_WT_ββα_K66A_ complex with peptide substrate LFP*.* **d**: FSC curves between two half maps without (green) and with (blue) mask. Resolutions estimated by FSC=0.143 criterion is marked. **e**: Cryo-EM reconstruction of PA26PANc-α_WT_ββα_K66A_ with substrate LFP at a resolution of 3.4 Å. **f** and **g**: Proximal and distal α-ring of the 20S CP from the reconstruction with LFP substrates. **h** and **i**: α-subunits from proximal and distal α-rings. Note that the N-termini of α-subunits from both α-rings in both reconstructions point upwards, consistent with an open gate conformation. **j** - **l**: Overlap of cryo-EM density of proximal (j), distal (k) α-subunit, and β-subunit with that of T20S alone.

**
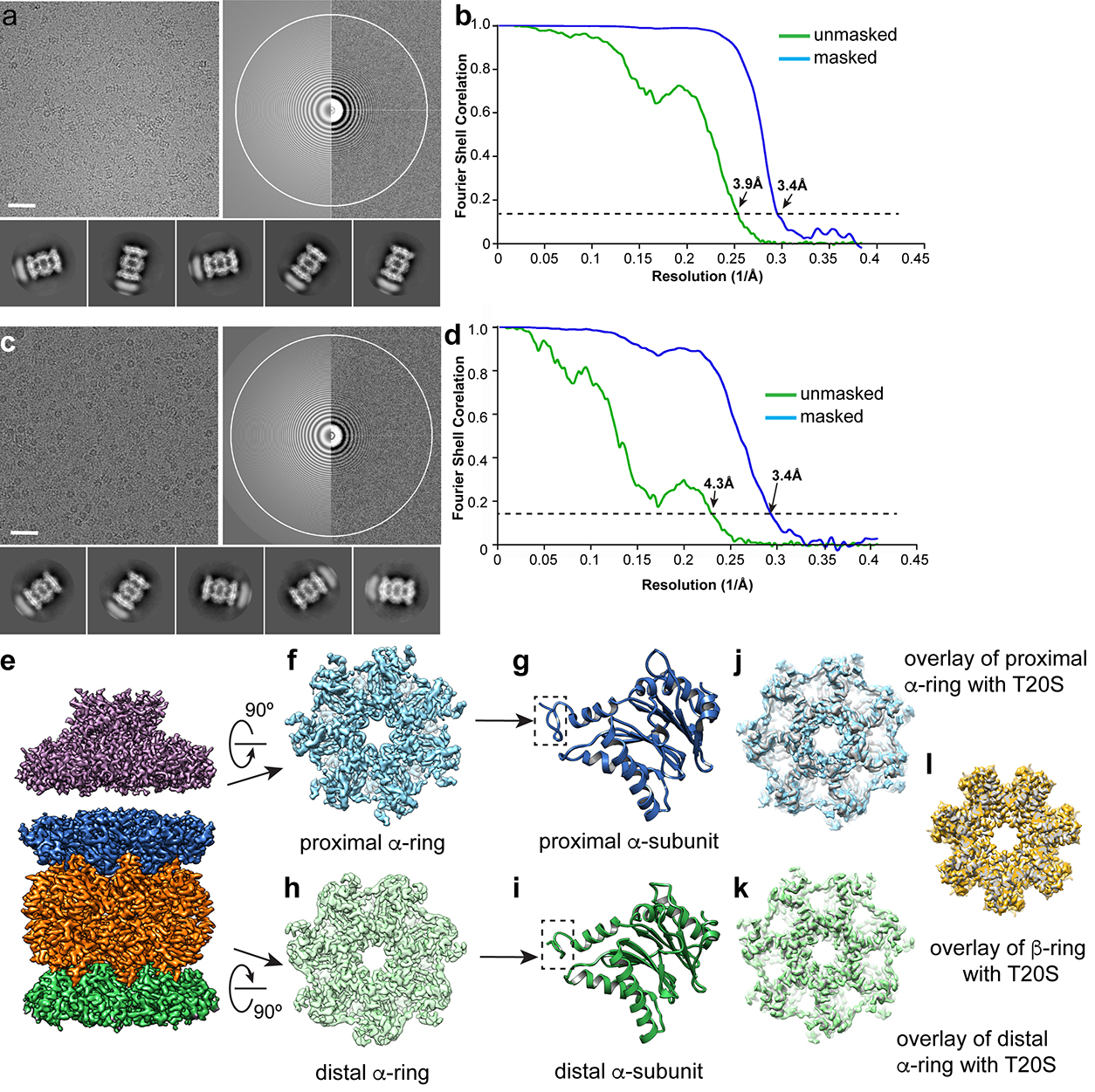
Supplementary Figure 8**. *Single particle cryo-EM of asymmetric* PAN-α_WT_ββα_K66A_ *complex without and with substrate GFP-ssrA*

**a**: A typical electron micrograph (scale bar 40 nm) of frozen hydrated PAN-α_WT_ββα_K66A_ complex without substrate (left), Fourier power spectrum calculated from the micrograph matched with simulated Thon rings (right), and typical 2D class averages of the complex. **b**: FSC curves between two half maps without (green) and with (blue) mask. Resolutions estimated by FSC=0.143 criterion is marked. **c**: A typical electron micrograph (scale bar 40 nm) of frozen hydrated PAN-α_WT_ββα_K66A_ complex with substrate GFP-ssrA. LFP*.* **d**: FSC curves between two half maps without (green) and with (blue) mask. Resolutions estimated by FSC=0.143 criterion is marked. **e**: Cryo-EM reconstruction of PANc-α_WT_ββα_K66A_ with substrate GFP-ssrA at a resolution of 3.4 Å. **f** and **g**: Proximal and distal α-ring of the 20S CP from the reconstruction with GFP-ssrA substrates. **h** and **i**: α-subunits from proximal and distal α-rings. Note that the N-termini of α-subunits from both α-rings in both reconstructions point upwards, consistent with an open gate conformation. **j** - **l**: Overlap of cryo-EM density of proximal (j), distal (k) α-subunit, and β-subunit (e) with that of T20S alone.

**
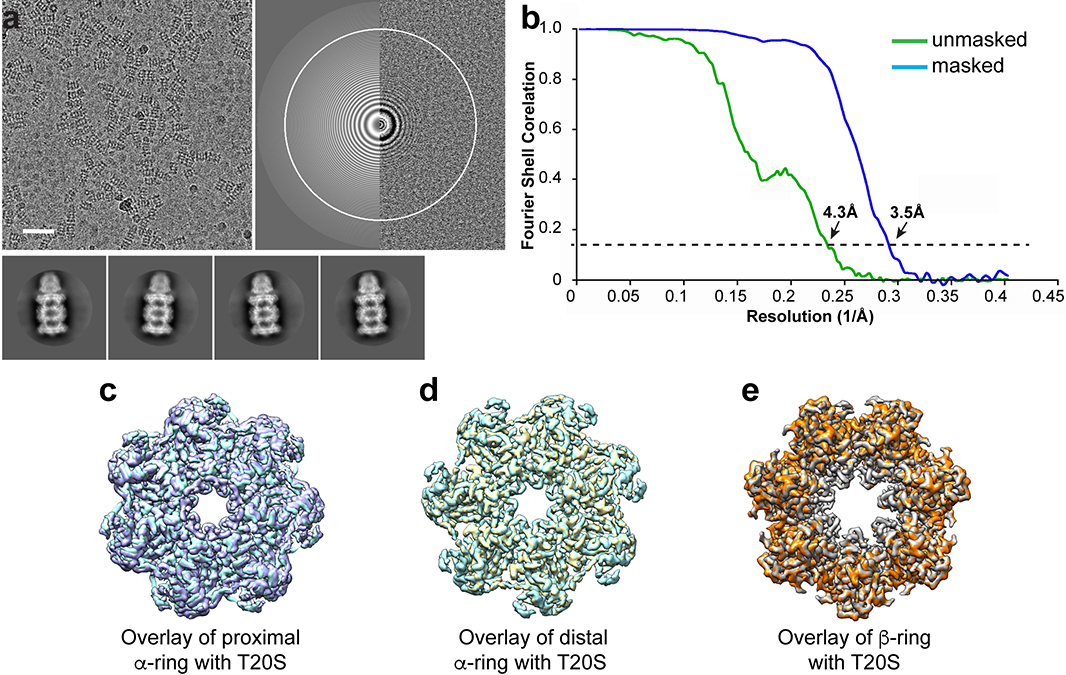
Supplementary Figure 9**. *Single particle cryo-EM of asymmetric* PA26-α_WT_ββα_K66A_ *complex*

**a**: A typical electron micrograph (scale bar 40 nm) of frozen hydrated PA26-α_WT_ββα_K66A_ complex without substrate (left), Fourier power spectrum calculated from the micrograph matched with simulated Thon rings (right), and typical 2D class averages of the complex. **b**: FSC curves between two half maps without (green) and with (blue) mask. Resolutions estimated by FSC=0.143 criterion is marked. **c** - **e**: Overlay of cryo-EM density of proximal (c), distal (d) α-subunit, and β-subunit (e) with that of T20S alone.

**
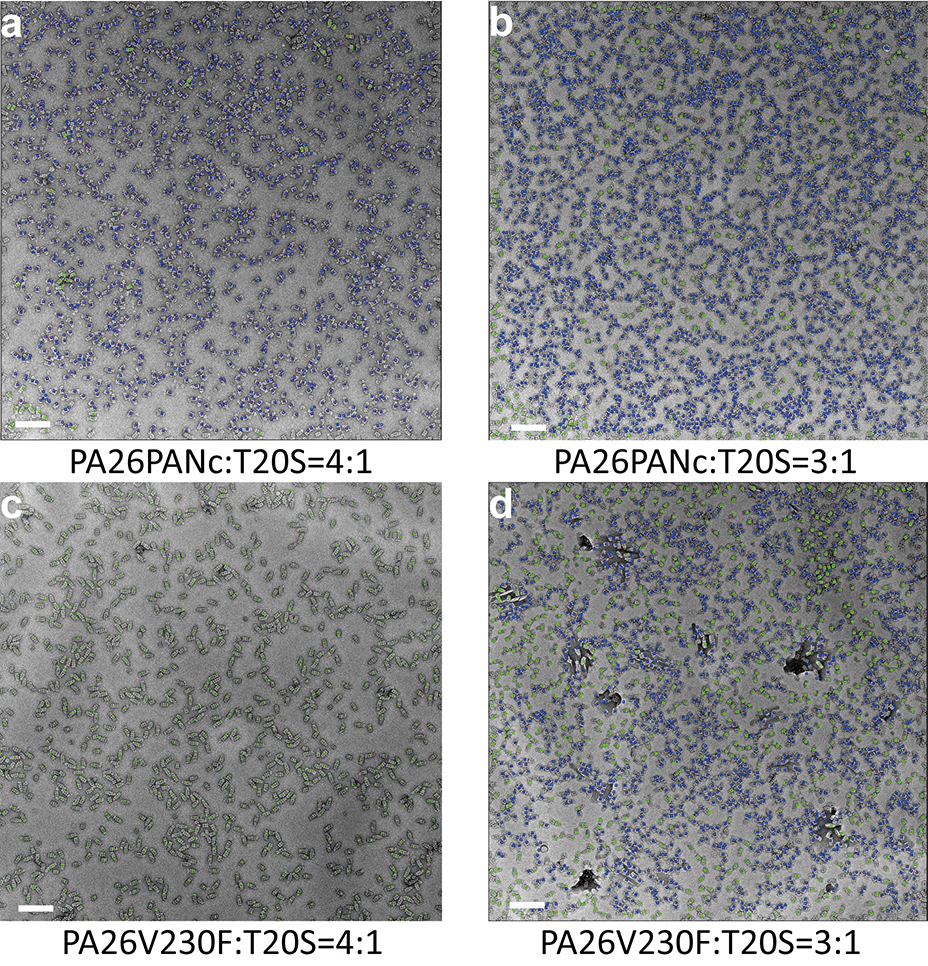
Supplementary Figure 10**. *Electron micrographs of negatively stained archaeal 20S proteasomal complexes*

Typical electron micrographs (scale bar 100 nm) of negatively stained PA26PANc in complex with wild type archaeal 20S CP in two different molar ratios, 4:1 (**a**) and 3:1 (**b**), and PA26 in complex with wild type archaeal 20S CP in two different molar ratios, 4:1 (**c**) and 3:1 (**d**).

**Supplementary Table 1** Expression vectors and plasmids used to express individual components of proteasomal subunits and activators.

| Gene | vector | Origin |
| --- | --- | --- |
| β∆_pro_-tev-6His | pET28a | This work |
| α_wt_-tev-6His/α_wt_-MBP | petDuet-1 | This work |
| α_R28L_-tev-6His/α_R28L_-MBP | petDuet-1 | This work |
| α_K66A_-tev-strep | pET28a | This work |
| 6His-PA26PANc | pET28a | Ref ^3^ |
| 6His-PA26V230F | pET28a | This work |
| 6His-PAN | pET15b | Ref ^3^ |
| β-tev-6His, α | pRSF | This paper |

**Supplementary Table 2** Summary of mutations used in this study and their functionality.

| mutations | subunit | Functional consequence of the mutation |
| --- | --- | --- |
| K66A | α-subunit | This mutation abolishes binding of activators, including both PA26 and PAN, to α-ring of T20S ^4^. |
| R28L | α-subunit | This point mutation enhances binding affinity of PAN to α-ring of T20S. It does not influence the gate-opening by PAN. |
| Δ2-8 | β-subunit | Peptide 2-8 is a propeptide that is autocleaved during assembling of CP. Its truncation is necessary to assemble the T20S *in vitro*. ^5^ |
| V230F | PA26 | To enhance the binding affinity of PA26 to α-ring of T20S ^6^ |
| E102A, PA26PANc | PA26 | E102A abolishes interaction between the PA26 activation loop with the reverse turn loop of T20S α-ring. Together with the C-terminal replacement of PANc, the chimeric activator activates the 20S CP in the same manner as PAN ^3^ |

**Supplementary Table 3** Microscopes and imaging conditions used cryo-EM data collections.

| Sample | microscope | voltage | Camera | Pixel size | Mag | Dose rate | Total dose |
| --- | --- | --- | --- | --- | --- | --- | --- |
| PA26PANc-T20S | Tecnai F20 | 200kV | K2 | 1.234Å/pix | 36K | 8e^-^/pix/s | 60e^-^/Å^2^ |
| PA26PANc-T20S/LFP | Talos Arctica | 200kV | K2 | 1.15Å/pix | 36K | 8e^-^/pix/s | 60e^-^/Å^2^ |
| PAN-T20S | Talos Arctica | 200kV | K3 | 1.08Å/pix | 36K | 33e^-^/pix/s | 60e^-^/Å^2^ |
| PAN-T20S/GFP | Polara | 300kV | K2 | 1.22Å/pix | 29K | 8e^-^/pix/s | 60e^-^/Å^2^ |
| PA26V230F-T20S | Talos Arctica | 200kV | K2 | 1.15Å/pix | 36K | 8e^-^/pix/s | 60e^-^/Å^2^ |

| Sample | Micrograph  Number | Particle  Number | Final  Resolution | Figures |
| --- | --- | --- | --- | --- |
| PA26PANc-T20S | 634 | 80251 | 2.87 Å | Supplementary Figure 7a |
| PA26PANc-T20S/LFP | 584 | 149626 | 3.46 Å | Supplementary Figure 7c |
| PAN-T20S | 1372 | 56754 | 3.41 Å | Supplementary Figure 8a |
| PAN-T20S w GFP | 1821 | 22773 | 3.43Å | Supplementary Figure 8c |
| PA26V230F-T20S | 719 | 77603 | 3.4 Å | Supplementary Figure 9a |
| T20S in vitro assembled | 51 | 3008 | 3.5 Å | Supplementary Figure 2a |
| Alpha doubly ring | 817 | 15812 | 3.7 Å | Supplementary Figure 1d |

**Supplementary Table 4** Vmax values measured for each concentration of different activators used in Supplementary Figure 4a – c.

| T20S: | 16nM | 16nM | 16nM | 16nM | 16nM | 16nM |
| --- | --- | --- | --- | --- | --- | --- |
| PA26PANc | 0nM | 16nM | 24nM | 32nM | 80nM | 160nM |
| Vmax | 86.05±10.71 | 267.2±33.46 | 343.7±47.84 | 448.6±73.94 | 534.3±86.19 | 525.4±70.12 |

| T20S: | 16nM | 16nM | 16nM | 16nM | 16nM | 16nM |
| --- | --- | --- | --- | --- | --- | --- |
| PA26V230F | 0nM | 16nM | 24nM | 32nM | 80nM | 160nM |
| Vmax | 64.89±5.177 | 203.4±8.852 | 237.6±18.33 | 249.1±30.18 | 486.5±40.9 | 426.2±35.27 |

| T20S | 16nM | 16nM | 16nM | 16nM | 16nM |
| --- | --- | --- | --- | --- | --- |
| PAN ATP | 0nM | 16nM | 24nM | 32nM | 80nM |
| Vmax | 156.7±15.82 | 334.9±48.47 | 251.4±50.01 | 540.5±63.85 | 452.4±58.09 |

|  |  | N2 | N1 | N0 | N | E2 | E1 | E0 | P | Q | F2=P^2^ | F1=2PQ | F0=Q^2^ |
| --- | --- | --- | --- | --- | --- | --- | --- | --- | --- | --- | --- | --- | --- |
| PA26PANc：T20S  =4:1 | Subset1 | 1334 | 3099 | 2852 | 7285 | 0.183 | 0.425 | 0.392 | 0.396 | 0.604 | 0.157 | 0.478 | 0.365 |
|  | Subset2 | 1883 | 2446 | 3292 | 7621 | 0.247 | 0.321 | 0.432 | 0.408 | 0.592 | 0.166 | 0.483 | 0.351 |
|  | Subset3 | 788 | 2222 | 2418 | 5428 | 0.145 | 0.409 | 0.445 | 0.350 | 0.650 | 0.122 | 0.454 | 0.423 |
|  | Total | 4005 | 7767 | 8562 | 20334 | 0.197 | 0.382 | 0.421 | 0.388 | 0.613 | 0.151 | 0.475 | 0.375 |
| PA26V230F：T20S  =4:1 | Subset1 | 2410 | 3220 | 640 | 5937 | 0.384 | 0.514 | 0.102 | 0.641 | 0.359 | 0.411 | 0.460 | 0.129 |
|  | Subset2 | 1945 | 2807 | 897 | 5649 | 0.344 | 0.497 | 0.159 | 0.593 | 0.407 | 0.351 | 0.483 | 0.166 |
|  | Subset3 | 2269 | 2564 | 696 | 5529 | 0.410 | 0.464 | 0.126 | 0.642 | 0.358 | 0.412 | 0.460 | 0.128 |
|  | Total | 6624 | 8591 | 2233 | 17448 | 0.380 | 0.492 | 0.128 | 0.626 | 0.374 | 0.392 | 0.468 | 0.140 |
| PA26PANc：T20S  =3:1 | Subset1 | 353 | 1403 | 6109 | 7865 | 0.045 | 0.178 | 0.777 | 0.134 | 0.866 | 0.018 | 0.232 | 0.750 |
|  | Subset2 | 426 | 1574 | 5228 | 7228 | 0.059 | 0.218 | 0.723 | 0.168 | 0.832 | 0.028 | 0.279 | 0.693 |
|  | Subset3 | 312 | 1646 | 5471 | 7429 | 0.042 | 0.222 | 0.736 | 0.153 | 0.847 | 0.023 | 0.259 | 0.718 |
|  | Total | 1091 | 4623 | 16808 | 22522 | 0.048 | 0.205 | 0.746 | 0.151 | 0.849 | 0.023 | 0.257 | 0.721 |
| PA26V230F：T20S  =3:1 | Subset1 | 1007 | 3934 | 3009 | 7950 | 0.127 | 0.495 | 0.378 | 0.374 | 0.626 | 0.140 | 0.468 | 0.392 |
|  | Subset2 | 720 | 2783 | 2443 | 5946 | 0.121 | 0.468 | 0.411 | 0.355 | 0.645 | 0.126 | 0.458 | 0.416 |
|  | Subset3 | 591 | 3003 | 2504 | 6098 | 0.097 | 0.492 | 0.411 | 0.343 | 0.657 | 0.118 | 0.451 | 0.431 |
|  | Total | 2318 | 9720 | 7956 | 19994 | 0.109 | 0.480 | 0.411 | 0.349 | 0.651 | 0.122 | 0.454 | 0.424 |

**Supplementary Table 5** Reproducibility of distributions of double, single and uncapped 20S

Micrographs of each sample are randomly divided into three groups with equal number of micrographs. Numbers of particles from each category, double, single and none, are determined from 2D classifications. The definitions of each symbol are listed below:

N2: number of 20S particle with both ends capped; N1: number of 20S particles with only one end capped; N0: number of 20S particles without capping; N: total number of particles; N = N0 + N1 + N2;

E2: percentage of double-capped 20S observed from the micrographs; E2 = N2/N; E1: percentage of single-capped 20S observed from the micrographs; E1 = N1/N; E0: percentage of non-capped 20S observed from the micrographs; E0 = N0/N;

P: probability of capping; P = (2 × E2 + E1)/2; Q: probability of non-capping; Q = (2 × E0 + E1)/ 2 = 1 – P;

Assuming there is no cooperativity between binding of activator to each end of 20S particle (null hypothesis):

F2: calculated percentage of 20S particles with both ends capped; F2 = P^2^; F1: calculated percentage of 20S particles with a singly cap; F1 = 2PQ; F0: calculated percentage of 20S particles without cap; F0 = Q^2^;

Calculated number of particles in each category under the null hypothesis:

A2: anticipated number of 20S with both ends capped; A2 = F2 × N; A1: anticipated number of 20S with one end capped; A1 = F1 × N;

A0: anticipated number of 20S without any cap; A0 = F0 × N;

**Supplementary Table 6** Analysis of distributions of double, single and uncapped 20S CP.

Sample: E2 E1 E0 P Q F2 F1 F0

PA26PANc:20S 0.192 0.385 0.422 0.385 0.615 0.148 0.472 0.379

(4:1) ±0.052 ±0.056 ±0.028 ±0.031 ±0.031 ±0.023 ±0.015 ±0.038

PA26PANc:20S 0.048 0.206 0.745 0.152 0.848 0.023 0.257 0.720

(3:1) ±0.009 ±0.024 ±0.028 ±0.017 ±0.017 ±0.005 ±0.024 ±0.029

PA26:20S 0.380 0.491 0.129 0.625 0.375 0.392 0.467 0.141

(4:1) ±0.033 ±0.025 ±0.028 ±0.028 ±0.028 ±0.035 ±0.013 ±0.022

PA26:20S 0.115 0.485 0.400 0.357 0.643 0.128 0.459 0.413

(3:1) ±0.016 ±0.015 ±0.019 ±0.016 ±0.016 ±0.011 ±0.009 ±0.020

Average and standard deviation are calculated from numbers obtained from each group, shown in Supplementary Table 4.

**References for supplementary figures and tables**

1 Huang, R., Perez, F. & Kay, L. E. Probing the cooperativity of Thermoplasma acidophilum proteasome core particle gating by NMR spectroscopy. *Proc Natl Acad Sci U S A* **114**, E9846-E9854, doi:10.1073/pnas.1712297114 (2017).

2 Li, X. *et al.* Electron counting and beam-induced motion correction enable near-atomic-resolution single-particle cryo-EM. *Nat Methods* **10**, 584-590 (2013).

3 Yu, Y. *et al.* Interactions of PAN's C-termini with archaeal 20S proteasome and implications for the eukaryotic proteasome-ATPase interactions. *EMBO J* **29**, 692-702 (2010).

4 Forster, A., Masters, E. I., Whitby, F. G., Robinson, H. & Hill, C. P. The 1.9 A structure of a proteasome-11S activator complex and implications for proteasome-PAN/PA700 interactions. *Mol Cell* **18**, 589-599 (2005).

5 Zwickl, P., Kleinz, J. & Baumeister, W. Critical elements in proteasome assembly. *Nat Struct Biol* **1**, 765-770 (1994).

6 Stadtmueller, B. M. *et al.* Structural models for interactions between the 20S proteasome and its PAN/19S activators. *J Biol Chem* **285**, 13-17 (2010).
