# Supplementary material for: Allosteric coupling between α-rings of the 20S proteasome
Source: Nat Commun. 2020 Sep 11;11:4580. doi: 10.1038/s41467-020-18415-7 (PMC7486400; doi:10.1038/s41467-020-18415-7)
Supplement: Supplementary file 3 — Reporting Summary [file 41467_2020_18415_MOESM3_ESM.pdf]

nature research

Corresponding author(s): Yifan Cheng and Philip Coffino

Last updated by author(s): Jul 27, 2020

Reporting Summary

Nature Research wishes to improve the reproducibility of the work that we publish. This form provides structure for consistency and transparency in reporting. For further information on Nature Research policies, see our Editorial Policies and the Editorial Policy Checklist.

Statistics

For all statistical analyses, confirm that the following items are present in the figure legend, table legend, main text, or Methods section.

n/a	Confirmed	
☐	☒	The exact sample size (n) for each experimental group/condition, given as a discrete number and unit of measurement
☐	☒	A statement on whether measurements were taken from distinct samples or whether the same sample was measured repeatedly
☐	☒	The statistical test(s) used AND whether they are one- or two-sided<br>*Only common tests should be described solely by name; describe more complex techniques in the Methods section.*
☒	☐	A description of all covariates tested
☒	☐	A description of any assumptions or corrections, such as tests of normality and adjustment for multiple comparisons
☒	☐	A full description of the statistical parameters including central tendency (e.g. means) or other basic estimates (e.g. regression coefficient) AND variation (e.g. standard deviation) or associated estimates of uncertainty (e.g. confidence intervals)
☐	☒	For null hypothesis testing, the test statistic (e.g. F, t, r) with confidence intervals, effect sizes, degrees of freedom and P value noted<br>*Give P values as exact values whenever suitable.*
☒	☐	For Bayesian analysis, information on the choice of priors and Markov chain Monte Carlo settings
☒	☐	For hierarchical and complex designs, identification of the appropriate level for tests and full reporting of outcomes
☒	☐	Estimates of effect sizes (e.g. Cohen's d, Pearson's r), indicating how they were calculated

Our web collection on statistics for biologists contains articles on many of the points above.

Software and code

Policy information about availability of computer code

Data collection	EM images are acquired using SerialEM3.8, followed by on-the-fly motion correction by MotionCor2.
Data analysis	Image analysis were performed following standard software: RELION1.4, Relion2.1, MotionCor2, GCTF, Gautomatch, cryoSPARC and cryoSPARC2 . Atomic model building is done with COOT, followed by iterative refinement with Phenix.real_space_refine. Figure preparation used Chimera and Pymol.

For manuscripts utilizing custom algorithms or software that are central to the research but not yet described in published literature, software must be made available to editors and reviewers. We strongly encourage code deposition in a community repository (e.g. GitHub). See the Nature Research guidelines for submitting code & software for further information.

Data

Policy information about availability of data

All manuscripts must include a data availability statement. This statement should provide the following information, where applicable:

- Accession codes, unique identifiers, or web links for publicly available datasets
- A list of figures that have associated raw data
- A description of any restrictions on data availability

Atomic models and cryo-EM density maps have been deposited to the Protein Data Bank and the Electron Microscopy Data Bank, under the accession numbers: PDB-6UTF, PDB-6UTG, PDB-6UTH, PDB-6UTI and PDB-6UTJ; and EMD-20877, EMD-20878, EMD-20879, EMD-20880 and EMD-20881, respectively.

Field-specific reporting

Please select the one below that is the best fit for your research. If you are not sure, read the appropriate sections before making your selection.

[x] Life sciences [ ] Behavioural & social sciences [ ] Ecological, evolutionary & environmental sciences

For a reference copy of the document with all sections, see nature.com/documents/nr-reporting-summary-flat.pdf

Life sciences study design

All studies must disclose on these points even when the disclosure is negative.

Sample size	A total of five dataset were collected to determine 5 structures in this study. The number of micrographs and particles in each dataset is presented in Supplementary Table 3
Data exclusions	No data is excluded
Replication	Biochemical experiments are triplicated. All repeated experiments were successfully carried out.
Randomization	Gold standard Fourier Shell Correlation method was used to estimate the resolution of cryo-EM structures, in which the dataset is split into two sets and refined independently. The process of splitting the dataset, odd and even, is considered random.<br>No other randomization is performed. For biochemical experiment, randomizations is not considered necessary.
Blinding	All experiments are not blind. Blindness is not standard procedure in structural studies.

Reporting for specific materials, systems and methods

We require information from authors about some types of materials, experimental systems and methods used in many studies. Here, indicate whether each material, system or method listed is relevant to your study. If you are not sure if a list item applies to your research, read the appropriate section before selecting a response.

Materials & experimental systems

n/a	Involved in the study
[ ]	[x] Antibodies
[x]	[ ] Eukaryotic cell lines
[x]	[ ] Palaeontology and archaeology
[x]	[ ] Animals and other organisms
[x]	[ ] Human research participants
[x]	[ ] Clinical data
[x]	[ ] Dual use research of concern

Methods

n/a	Involved in the study
[x]	[ ] ChIP-seq
[x]	[ ] Flow cytometry
[x]	[ ] MRI-based neuroimaging

Antibodies

Antibodies used	Anti-GPF monoclonal antibody GF28R (ThermoFisher Scientific, catalog # MA5-15256) was used for western blot
Validation	No further validation is performed.
