# Supplementary material for: Allosteric coupling between α-rings of the 20S proteasome
Source: Nat Commun. 2020 Sep 11;11:4580. doi: 10.1038/s41467-020-18415-7 (PMC7486400; doi:10.1038/s41467-020-18415-7)
Supplement: Supplementary file 2 — Peer Review File [file 41467_2020_18415_MOESM2_ESM.pdf]

Comments and response to Reviewer #1

Reviewers first comments
Author's first reply

Reviewer #1 (Remarks to the Author):

The main motivation of this manuscript is to investigate how a diverse population of the proteasome particles are assembled in general, and more specifically, whether a doubly-ATPase-capped proteasome functions similarly to or differently from a singly-ATPase-capped counterpart. Through a series of cleverly designed biochemical and structural experiments, the authors found – rather unexpectedly - that the singly-ATPase-capped assembly functions with essentially the same efficiency as the doubly-ATPase capped version. They went on to disclose that the high efficiency of the singly-capped is achieved via an allosteric mechanism: gate opening at one end of the 20S core particle by a HbXY-motif containing activator triggers the rearrangement of the N-termini of the alpha-ring, hence the opening of the substrate gate, of the opposite end. The allostery of the 20S CP is found to be dependent on the HbXY motif present in the PAN ATPase (or the Rpt1-6 ATPases in eukaryotes) but is absent in the non-ATPase activators. This finding explains the fact that 20S exhibits allosteric activation only with the ATPases but not with non-ATPases such as the PA26 ring. Finally, the authors discovered the cooperative assembly of the doubly-capped 20S CP by the HbXY-containing activator only but not by non-HbXY motif. This discovery lends a strong support to the proposed allosteric mechanism. Overall, proteasome is one of the most central molecular machines in life, regulating numerous cellular processes. The main conclusion of the manuscript is novel, supported by experimental evidences as presented. The work appears to be a good candidate for publication in Nat Communs.

We appreciate this general comment, and will address all specific comments, major or minor, in the revision.

The manuscript needs to be revised to address our comments and concerns as listed below.

1. Line 72: The two point-mutations affecting activator binding have been described previously. The authors are simply using these mutations as tools to probe their question. Therefore, it is better that the details about the mutations are introduced here as background and relevant literature cited here, not in the Results section. By the same token, E102A and V203F mutations in PA26 should also be introduced in Introduction.

Indeed, the K66A mutation is well characterized previously and is a critical mutation we used to assemble asymmetric 20S CP. Following this suggestion, we revised the Introduction by including the K66A mutation and proper citation in the Introduction. Regarding the E102A and V230F mutations in PA26, since it requires a more detailed explanation, particularly E102A, and it is part of the our experimental design, we prefer

to keep it in the first section of Results, where we described the overall design of our experimental approach.

2. Line 98 (Supplementary Figure 1K): The in vivo 20S is over two times more active than the in vitro 20S, and the activity of 20S with activator is ~3 times higher than the in vitro version. Therefore, saying … its proteolytic activity level is "slightly" lower than that of the native 20S CP … is inaccurate. It may be more adequate to state that the activity measurements demonstrate the in vitro assembled 20S CP is functional or active.

We agree and revised the text.

3. Supplementary Figure 1K lacks controls. For both 20S CP in vivo and 20S CP in vitro, florescence of samples without substrate should be measured and provided.

The requested control has already been done. The value reported in Supplementary Figure 1K are normalized after subtracting the florescence reading from the sample without substrate. We have revised the Methods section to make this clear.

4. Line 120: Add one sentence to explain why K66A mutation impairs binding – and this known fact should be moved to Introduction, as noted in #1.

We have revised the text to provide an explanation of K66A, and cited appropriate references.

5. Line 245: This result (Figure 3) – should be Figure 4.

Corrected in the revised manuscript.

6. Line 269: … singly capped asymmetric CP (Figure 5B) … – should be Figure 5g.

Corrected in the revised manuscript.

7. Line 293. Supplementary Table 3 and 4. Not found in the submission. Perhaps all information has been combined in Supplementary Table 1?

We apologize that the Supplementary Table 3 and 4 were missing from our submission. These are now included in the revision.

8. Fig 2c-d: The amount of GFP substrate in (c) could be roughly quantified and shown as a separate plot. In panel d Western blot image, did the WM markers cross-react with the antibody? If not, the panel including the marker lane may be a composite image? If this is the case, please leave some white space between the spliced images – the markers and the substrate lanes.

We thank the reviewer for this suggestion. While we prefer to keep the original gel image as Figure 2c, we now quantified the GFP substrate in this panel and shown as a

separate plot as Supplementary Figure 2. As for the panel d, the WM markers do not cross-react with the antibody. Instead, the MW proteins were pre-stained before western blot with antibody. Therefore, this is not a composite image. We now made this clear in both Figure legend and in The Methods section.

9. Fig 5: Please add atomic model comparison of the alpha-rings, just like in Fig 3c, g and Fig. 4c, g.

We have revised figure 5.

10. The non-allostery of the gate opening observed here (and elsewhere) with PA26 and the archaeal 20S CP is consistent with the previous observations of the non-allosteric behavior of an actinobacterial 20S CP by a non-ATPase activator (PMID 27001842, PMID 29414791). This is relevant and should perhaps be cited here because both are prokaryotic systems.

Very good suggestion. We revised the Discussion section and added these two references.

Comments and response to Reviewer #2

Reviewers first comments
Reviewers first reply
Reviewers second reply
Author's first reply
Authors second reply
[Authors third reply]

We thank the Reviewer for his/her time and efforts in providing valuable feedback to our revised manuscript. While the Reviewer disagreed with some of our responses in the previous revision, his/her comments and suggestions have become more specific. Following these suggestions and comments, we have now carried out most of the control experiments suggested by the reviewer. Fortunately, we were able to complete almost all new experiments before lab closure due to COVID-19. Indeed, these control experiments enable us to describe our experimental conditions more precisely, thus strengthening our conclusions. We would like to express our sincere appreciation to the Reviewer and apologize that we did not grasp the meaning of some of his/her comments in the previous round of revision or overlooked some aspects of our data presented in the previous version of the manuscript. We have now revised the manuscript further with new data incorporated from these additional control experiments. We believe the revised manuscript is significantly improved. In the following, we provide our point-to-point response to all comments from the reviewer.

Reviewer #2.

The reviewers now provide the missing supplemental figures, and have addressed a couple concerns that were addressed previously. However, in short, the authors ignored many of the

requests to validate their findings with supporting experimental approaches and they did not address reasonable alternative hypothesis that were raised. It is this reviewer's opinion that the results shown do not rigorously support the conclusions that are drawn. Please see the point by point responses below in green.

We apologize for not fully addressing previous comments. While we still disagree with a few points raised by the reviewer, with respect, we have now carried out some additional control experiments to address most of the comments raised here.

[To make it easier to keep track of questions/comments and responses from multiple rounds of revisions, our third cycle of responses are in red text and bracketed within square parentheses, and they are inserted into the second reply of Reviewer 2, which is in bold green text.]

Reviewer #2 (Remarks to the Author):

The manuscript entitled "Allosteric coupling…" by Yu et. al. presents a potentially interesting and novel finding that HbYX containing proteasomal regulatory particles (RPs) allosterically alter the alpha ring on the opposite side of the 20S core particle (CP) from where they bind. The study is done with 20S from archaea. The authors created a novel and ingenious methodology to generate CP's with different alpha rings, one capable of binding to RP's and the other defective in RP binding. This allowed the generation of homogeneous single bound, RP-CP's, and doubly bond, RP-CP-RP complexes. The authors studied these single and double cap structures biochemically and via cryo-EM.

In short, the biochemical studies were not done very carefully, and were not conclusive since some likely alternative hypothesis were not addressed by the experiments that were done. Important controls were also not included. In addition, the structure data that was presented was weak, based on very small changes in the Cryo-EM data sets, and did not contain critical control structures to compare the open and closed channel states with the asymmetric mutants that were used.

We would like to point out the statements made here are unjustified. In the following, we provide our point-by-point response to each critique raised by this reviewer.

In conclusion, the results that are presented here do not support the conclusions that were made in this manuscript. Major and minor problems and further details to these general comments are listed below.

Major problems:

1) The conclusion from Fig 2A is that the RP (pa26panc) in the single and double cap forms has similar activity, but it's very clear that the double cap form show ~20% more activity than the single cap form, especially at the 7 and 15mM substrate concentrations. This also looks to be significant just from eyeballing the error bars. This is in conflict with the authors conclusions.

With respect, we would like to point out that this assessment is incorrect. When comparing the activities between the two different proteasome complexes (double verses single capped), the most important parameter is the Vmax, not individual data points. As listed in the table (Figure 2b), the Vmax of doubly capped proteasomes is 199.1+/-19.1, and that of singly capped proteasomes is 230.5+/-17.5. The two activities are identical within experimental error. Thus, it

is correct to state that the two different proteasomes have similar activities. Even to compare specific data points, such as 7 and 15 mM substrates, the changes from without activator to with activator bound lies within the error bar for two different activators and 20S proteasomes. Therefore, our conclusion about this specific experiment is correct.

I disagree, when considering the use of peptide entry as a proxy for monitoring substrate entry rates, any one concentration of substrate is just as valid as the vmax. In fact, high concentrations are more likely to mask gate-opening effects because at high concentrations, substrate hydrolysis may become rate limiting (rather than substrate entry). Substrate entry is a measure of access, not a measure of affinity (as there is no binding site at the gate. In this case, the role of the gate is to limit the internal concentration of peptide. In addition, the authors argue that their Vmax data indicates no –significant difference however, they cannot use this to argue there is not a difference, only that they their results cannot predict a difference. My original concerns still stand, though this concern is more minor compared to others below.

If we look at the Figure 2a alone, indeed, there is a small difference (~15% in Vmax, 230 vs. 200, or in individual data points) between peptidase activity of 20S proteasomes singly and doubly capped with PA26PANc. The disagreement between our interpretation and that of the reviewer's is whether this difference is significant enough to conclude that peptidase activity of doubly capped 20S is higher (significant) or similar (not significant) compared to that of 20S singly capped by PA26PANc. If this was the only data, it would indeed be difficult to judge whether a 15% difference (smaller if considering the error bars) is sufficient to alter our conclusions.

By comparison, we also take into consideration the data shown in Figure 5g. This experiment is similar in design to that of Figure 2a, the difference being that here we are using the regulatory complex PA26, which does not have the C-terminal HbYX motif, in place of PA26PANc, which has the HbYX motif. With PA26 the difference of Vmax (or individual data point) between doubly and singly capped 20S is ~100% (800 vs. 400). With this comparison, we concluded that the difference shown in Figure 2A, using a regulatory complex with the HbYX motif, is not significant. In our view, this interpretation is reasonable and can be further explained by (and is consistent with) the structural data: PA26PANc allosterically opens the distal gate, and thus confers no difference (or insignificant difference) in the peptidase activities between singly and doubly capped 20S; PA26 does not allosterically open the distal gate. Thus, the peptidase activity of 20S doubly capped by PA26 is significantly higher, two-fold, than that of a singly capped one. In a subsequent comment, the Reviewer provided a completely opposite explanation, with which we disagree, and we provide below our rebuttal to that alternative explanation.

We may not have explained this clearly in our initial response, and we apologize for that lack of clarity.

The concern raised in the prior review (green) was not addressed. Analysis of only the Vmax is irrelevant, and all concentrations of substrate are relevant when looking at catalytic chamber access. [We disagree. Our goal is to compare the proteolytic activities between singly and doubly capped 20S CP. Therefore, we determined the dependence of activity at a broad range of substrate concentrations. This accomplishes several things. It confirms that the proteasome complexes, as enzymes, show a good approximation to Michaelis Menten kinetics. It yields directly the kinetic parameters, k_m and Vmax. Vmax is the most appropriate parameter for comparisons of activity among the diverse complexes.] **In addition, why did the authors do a substrate dose response? This doesn't make sense and adds**

little to the data. A dose response with the activator, would have made more sense. This experiment would allow one to determine the complex saturation level, which would have been far more informative and useful (the authors partially do this experiment (see below)). [A dose response with the activator measures the saturation of capping. We have done these experiments as controls to provide estimations of the fraction of 20S CP capped with activators, as shown in Supplementary Figure 4. The dose response of activator, however, does not provide comparison of maximum proteolytic activities between singly and doubly capped 20S CP. This requires determination of the dependence of activity on substrate concentration, as we describe immediately above.] **As mentioned in prior reviews, there is no evidence here that these complexes are actually saturated PA26-20S complexes, which is where the analysis needs to be done to be conclusive.** [The extent of saturation of CP by RP is a recurrent question from the reviewer. First, we did provide experimental estimations of saturation in the second revision, in terms of capping fraction (Supplementary Figure 4). Second, saturation is NOT a requirement for adequate analysis. We do not need to achieve saturation to get meaningful data, just sufficient capping to generate data clean enough to determine the extent of a capping effect, if present. At sub-saturating [RP], with saturation S, S between 0 and 1, there is a population present of uncapped complexes. Under these conditions, we are comparing experimental values of [uncapped] versus [capped X S] + [uncapped X (1-S)]. If activity values of uncapped and capped are different enough and S is sufficiently close to 1.0, we can get informative data. Those conditions are met in our experiments. S does not need to be ~1.0.] **In addition, a 5μM Km for these peptides must be wrong. The best peptides for the proteasome that we have (e.g. LLVY-AMC) have a Km that is >10mM.** [This is incorrect. There are many examples from the literature showing a K_m of LLVY-AMC in the range of μM, not mM. For example, Nat. Commun. 7:10963 (2016) doi: 10.1038/ncomms10963 fig. 2E shows a Km for LLVY of 90-100 uM; Molecular Cell: 4, 395, (1999), fig. 2C control, shows an apparent K_m in the 10-20 μM range.] **The type of longer quenched peptide that is used here is almost certainly precipitating or aggregating which is causing the flattening of the curve (not active site saturation). This is a common problem for the LFP peptide substrate and is a well-known issue.** [This is incorrect. Reviewer did not provide any citation for problems of LFP solubility in the concentration range we use. Instead, 10μM LFP was used in previous studies without any problem (Smith, et al. Molecular Cell 20, 687 (2005); Smith, et al. Molecular Cell 27, 731 (2007)). Furthermore, the hyperbolic dependence of peptidase activity on [LFP] we observe is consistent with Michaelis Menten active site saturation and inconsistent with solubility deffects.] **This can be seen in some of the data as a decreasing Vo at higher concentration in several of the data sets, especially those that have low degradation rates (allowing more aggregation at high concentrations), suggesting substrate precipitation causing the curves to flatten.** [It is not clear what is described here in our data as decreasing activity at high [LFP]. We are puzzled.] **{I will address the alternative explanation below when discussed by the authors.}**

2) The authors seem to assume that opening 2 gates would result in twice the rate of peptide entry and thus twice the rate of substrate cleavage, but this may not be true, and is not a trivial assumption. If the authors are operating under this assumption, which they appear to be, they should show the maths that support this assumption. It's not expected that doubling the surface area of entry should be linear with diffusion entry rate. (see Z. Schuss, A. Singer, and D. Holcman PNAS October 9, 2007). E.g. if opening one side of the proteasome allows the internal compartment to go to equilibrium with the external compartment, then there is no reason to expect that opening a second gate should increase peptide cleavage rate.

We did not make the assumption that two open gates would confer a higher peptidase activity compared to one open gate. This was a hypothesis to be tested by the experiments described.

The observed results, briefly, were that the outcome depended on whether the regulatory complex cap did or did not have a C-terminal HbYX motif. We found that for a cap with the motif, one cap (asymmetric case) versus two (symmetric case) conferred similar activity. For a cap without that motif the proteasome with two caps was about twice as active as the proteasome with one cap for unfolded peptide substrates. Exploring the implications of that finding- different activities conferred by two types of cap- led to the structural studies demonstrating distal gate opening in an asymmetric proteasome with one cap that contains the HbYX motif.

The authors did not address the original concern, if opening one gate allows the peptide's in the buffer to go to equilibrium with the 20S internal chamber than opening of a second gate should have NO effect on peptide hydrolysis rates.

Indeed, once the peptidase activities reach maximum, an equilibrium is reached. The rate limiting step can be either gate entry or proteolysis. Assuming that proteolysis is the rate determining factor, as the reviewer suggested, we should see no difference in Vmax between 20S singly and doubly capped with PA26 (Figure 5g). Again, by looking at the results shown in both Figures 2a and 5g together, we can conclude that the entrance of peptides into 20S degradation chamber is the rate limit step.

This is incorrect. Take the hypothetical instance of single or double caps having the same activity, would that imply that a single cap complex has a gate opened at both ends, or that only the double cap has a gate opened at both ends? If opening of one gate gives you maximal substrate entry and thus hydrolysis, this experiment cannot distinguish between these two alternative explanations of this data (this is why two different models were proposed before). While it's attractive to suggest that the PA26 data (5g) negates one of the PA26-PANc models is specious, as this is a different complex, that can act differently on the proteasome. [We are very puzzled by this statement. It is our finding that for PAN26PANc a singly capped 20S CP has the same peptidase activity as a doubly capped one, consistent with both gates being open. And that for PA26, the activity of singly capped 20S CP is about half of the doubly capped, consistent with a single gate, the proximal one, being open by PA26. The logic presented in our study is very clear.] **Perhaps PA26 acts on the proteolytic sites of the proteasome and activates them in some way.** [There is no evidence or literature that PA26 directly acts on the proteolytic sites of proteasomes, nor any plausible hypothesis suggesting that it does. It is also beyond the scope of this study to investigate the possibility that PA26 acts directly on the proteolytic site.] **The issues described above (Substrate precipitation, improper dose response experiment (should be PA26 dose response), low 20S activation by PA26, etc. prevent a rigorous conclusion from being made regarding the PA26-20S experiments. The experiments showing that the authors are actually using saturating amounts of PA26 would go far to helping show this, but they have not chosen to do these experiment.** [The question concerning saturation is answered above.]

Addition of regulators can affect peptide hydrolysis is many ways, including affecting the proteolytic mechanism, affecting substrate entry, and affecting free concentration of substrate. Any of these things could be responsible for the affects that are seen. A simple experiment with a 20 proteasome with an N-terminal deletion (open channel mutant) at one or both ends of the 20S would demonstrate the expected effects of opening 1 or both alpha rings in the 20S. This would allow the authors to rule out these other alternative hypothesis.

Indeed, an ideal experiment would be to compare the peptidase activities of a 20S proteasome with a single gate deletion versus one with a double gate deletion, so that the influence of gating by activator can be ruled out. This was our initial intent. Unfortunately, we were unable to generate in vitro assembled 20S proteasomes with either one or both gates deleted. Expression of α-subunit alone produces a double α-ring (Figure 1a, and Supplementary Figure 1c – g). Incubating with β-subunit under the right buffer condition separates the double α-ring so that fully intact 20S proteasomes can be assembled. Unfortunately, for unknown reasons, separation of double α-rings with gate deleted 20S is not observed, precluding the formation of intact gate-deleted 20S proteasomes. Thus, we were unable to generate any in vitro assembled 20S proteasome with gate deletion mutations.

We therefore used an alternative approach, by comparing the extent of activation by a regulatory complex with that conferred by a double gate deletion. We compared peptidase activity among in vivo produced 20S proteasomes with a double gate deletion (Supplementary Figure 1k bar 3) versus wild type 20S (with intact gates) activated by PA26PANc (Supplementary Figure 1k bar 2). Briefly, 10uM LFP fluorogenic substrate was incubated for 30 minutes with 16nM 20S with the double gate deletion, or with 16nM wild type 20S with 112nM PA26PANc as the gate-opening activator. As a control, 10uM LFP was incubated with 16nM wild type 20S without activator (Supplementary Figure 1k bar 1). Using the same method as in early publications, peptidase activity was determined from the rate of increase of florescence generated by hydrolysis of the peptide LFP substrate. Based on direct counting of complex compositions using negative stain EM we estimated that with wild type 20S with activator PA26PANc, ~86% of CP are doubly capped by, ~10% are singly capped and ~5% are uncapped. The data supporting this conclusion are shown in Supplementary Figure 4.

Thanks for providing this data in a clear manner, however it seems irrelevant to the prior concern. Though, the gate deletion mutant is not very activated with respects to the WT, 10-20 fold is more common, but this is minor. Perhaps the author could do a Dose response with LFP with these two 20S complex, and choose the LFP concentration that provides the larges fold difference in activity, this would be a better way to choose and substrate concentration that best reports on the state of the 20S gate, given LFP's precipitation and aggregation issues at higher concentrations. [10μM LFP is used, which is the same substrate concentration used in previous studies cited above. As described above, we remain puzzled by repeated mentions of LFP solubility problems at the concentrations used in the experiments reported here.]

As described in the response to point 6, biochemical experiments also support the conclusion that capping experiments were performed under conditions of near saturation of CP. The result was that peptidase activities of 20S proteasomes with near-saturating activator or with gate deletion are essentially the same. These data are now included in the updated Supplementary Figure 1k. From this experiment, we conclude that the gate opening by activator does not interfere with peptidase activities of 20S proteasomes, and gate-dependent entry is instead rate-determining.

I appreciate that the authors now show the saturation experiments for PA26-PANc, PA26V230F, and PAN on the T20S. However, these experiments only provide half of the information that is needed. These saturation experiments need to be done with the T20S, then again with the K66A mutants. This will allow them to conclude, under saturating

conditions (for both different T20S complexes), what the Vmax differences is, in the single and double caped states in a rigorous manner. [Please see our previous comment describing why saturation of CP by RP is not a necessary condition. Nonetheless, both cryo-EM and negative stain EM data and biochemical data support the conclusion that saturation is adequate in these experiments. We are also puzzled by the request for RP saturation binding experiments using 20S CP with the K66A mutation. That mutation prevents any RP activator binding.] **In addition, the experiments appear to have only been done in singlicate, so no standard deviations are given, and no statistics or standard errors for Km, or Vmax are given for the fit which is required to interpret the quality of the fit the data, and any conclusions that can be made from it.** [This is incorrect. The dose response experiments shown in Supplementary Figure 4a, b and c are triplicated with error bars in each data point. The Vmax and Km shown in Supplementary Figure 4g, h and I were derived from a, b and c.]

It appears that the Vmax values are used for this plot (based on those in the table according to the legend), but based on the assumption made above that this substrate appears to be aggregating or precipitating, then this would provide completely erroneous data. [We disagree, as fully aired above. This comment is based on undocumented assumptions about peptide substrate precipitation problems.] **These regulator dose response curves should be generated with an intermediate level of peptide, that is not in the precipitation concentration (pseudo-saturation concentration >10microM). E.g. 5-10microM would be more ideal, and would more accurately report on gate state condition, and thus would allow for an accurate assessment of fold change of activity of the double and single cap complexes.** [This is incorrect. The dose response curve needs to be carried out at a range of substrate concentrations. The data points used are 2.5, 5, 7.5, 10, 15, 20 and 25µM. The hyperbolic shape of saturation curves suggests that there is no substrate precipitation.]

Another major problem seen with this data is that the saturation phase for PA26VF curve is generated by a single point (sup fig 4), and the line fit statistics must be terrible. In addition, the PAN dose response data points are all over the place, and fitting a line to that data is completely pointless. If the authors simply paid attention to the confidence intervals in the fits, [We do agree that the data for PAN are weak, and provide only a rough indication of the extent of capping. This experiment would have been repeated if pandemic lab closure had not supervened. For the other two RPs the data support the stated conclusions.] **it would be obvious that these fits and saturation data are not useful, and tell us nothing regarding the saturation of these complexes.** [We disagree completely.] **In addition no statistics for these fits are given.** [We have now added statistics to Supplementary Figure 4a-f.]

Minor: The legend of sup fig 4 suggests that the y axis provides fold information this is incorrect and should be reworded. Also the figure call outs in this figure are all mislabeled. In addition, the method of starting the fit with the first concentration of the regulator, and subtracting the no regulator condition to graph the data is unorthodox, misleading and confusing. The first point (zero regulator) should show the actual V value. [We are determining the effect of RP binding, and therefore the no-RP condition constitutes the background for such determination. The absolute value of activity with no RP present is shown in sup. fig. 4, e-g, thus giving full information regarding the experimental data. As displayed in i-k, plots with background subtracted are used for hyperbolic fitting and determination of kinetic parameters.]

3) The experiments looking at PAN dependent degradation of GFP-ssrA do not account for the ability of the CP to degrade unfolded GFP. PAN is in excess in these experiments, so there is an appreciable amount of free/unbound PAN in this solution, which is capable of unfolding GFP-ssrA on it's own. This unfolded GFP can be readily degraded by the unbound CP, or even by the Free end of a CP. Since these experiments contain the same amount of PAN (only difference is single versus double cap CPs), than it could be expected that one would see similar amounts of degradation, especially since unfolding is the rate limiting step. The contribution of this possibility to GFP degradation could be assessed with the K66A mutants and PAN.

Again, we respectfully disagree with this comment. GFP rapidly refolds. Therefore, trans unfolding by PAN and subsequent degradation by free 20S will not happen, since free 20S have closed gates that prevent degradation of large substrates without activator. Even if it happens at all, it is very inefficient when compared with degradation by 20S physically coupled to the PAN unfoldase cap. There is a wealth of literature supporting this conclusion. See for example 10.1073/pnas.97.16.8898 Proc Natl Acad Sci U S A. 2000 Aug 1; 97(16): 8898–8903, figure 4.

If the authors know that "GFP rapidly refolds", too quickly for 20S to degrade it, and that "it is very inefficient" then they should show the data. The authors have the K66A 20S which blocks regulator binding, a control experiment with this mutant and PAN and GFP would demonstrate the capacity of the 20S to degrade unfolded GFP without association. The literature is full of experiments demonstrating that the 20S (especially the archaeal 20S) is an excellent protease for unfolded polypeptides; this a reasonable expectation, and addressing it is necessary for a rigorous conclusion.

We have now performed the suggested control experiment. We observed degradation of GFPssrA when it is incubated with wild type 20S with PAN and ATP, but no degradation when it is incubated with 20S-K66A with PAN and ATP. As further controls, no degradation was observed when GFPssrA is incubated with PAN + ATP without 20S, with uncapped wild type 20S or uncapped 20S-K66A without PAN. This control experiment confirms our assumption that degradation of GFPssrA is only carried out by the 20S-PAN complex with ATP, but not by free PAN or free 20S. We thank the reviewer for persuading us to perform this experiment, and the data are now provided as Supplementary Figure 5b.

I appreciate that the reviewers have now done this experiment, but there are some serious concerns seen in this data. For the 20S-K66A + PAN + ATP experiment, GFP-ssra amounts are higher at 5 and 15 min compared to the zero min control! Even more concerning is that the T20S, and T20SK66A controls both show substantial GFP-ssrA degradation at 30 and 60 min. Is it possible that there are contaminating ATPase (ClpX) in these preps? What most concerning is that this contaminating activity seen in the 20S only controls is not seen in the 20SK66A + PAN samples? Leaving out ATP would explain this result? [The question here is whether PAN and CP must be physically coupled to achieve GFP degradation. This question has been thoroughly answered in related experimental contexts, and is regarded as a settled matter- the answer is yes, they must be coupled. Nonetheless, we did the experiment, with all the right controls. The answer is, again, yes. Western blotting can be made quantitative by replicates, band quantitation when these are subsaturating in intensity, and other well-understood techniques. However, as generally performed, blotting is a semi-quantitative method, subject to potential artefacts of lane loading, locally incomplete transfer, non-uniformity of development, etc. The data shown here answer the question posed fully. Minor anomalies as described are largely due to loading differences among the lanes, as can be

seen be looking at the loading control bands. That is why loading controls are typically shown in Western blots. ClpX contamination (which is not a protease but an ATPase) is a rather exotic possibility, given the multi-step purification used for the recombinant proteins. Perhaps dandruff protease is a more plausible contaminant (Sing et al, Retention of Corneodesmosomes and Increased Expression of Protease Inhibitors in Dandruff, doi: 10.1111/bjd.13111). It does not make sense to repeat the experiment multiple times for a cosmetically perfect result. The important point is that the question asked by the reviewer is fully addressed here.]

Nevertheless, the experiments with the GFP substrate don't seem to add much to this model or manuscript. [We agree these experiments are redundant. They were performed in response to the specific insistence of this reviewer.] **Substrate unfolding is rate limiting, so if you have 1 PAN bound, you expect to see ½ the degradation rate of having 2 bound. This suggests that the K66A mutant functions as is expected, but says nothing about the allostery between alpha rings.** [This is an interesting question, to which we can provide only a speculative answer. There may be mechanisms that constrain both ATPases of a symmetric proteasome from being simultaneously active as unfoldases. In eukaryotic proteasomes, unfolding cycles of structurally stable proteins are associated with large iterative conformational changes of the 19S ATPase. Such changes in the *cis* ATPase could provide an allosteric signal that constrains activity of the t*rans* ATPase. An actively unfolding ATPase may thus behave differently from one that is simply bound and acting as a more passive gate opener. In this scenario, two copies of the RP ATPase (compared to one) would confer double peptidase activity, but not double unfolding and protease activity. The GroEL−GroES chaperonin cage is an example of a complex with such a functional constraint. GroEL can accept an active cis GroES cap at one of its two ends, but not at both simultaneously (Hartl et al., Nature 475:324, 2011).]

4) All single cap CP-RP complexes in the study contain a K66A mutation at the distal CP end, which is the end that is presumed to be allosterically opened. Could this K66A alpha ring inherently display different gate conformations (e.g. open), than the WT alpha ring? If so this would explain the result.

We have determined a structure of singly capped 20S-PA26. In such a structure, the distal alpha ring contains the K66A mutation and the gate remains closed. Therefore, the point mutation K66A does not influence the gate conformation. This is also consistent with many previous published studies, such as Forster, A., et al (2005). Molecular Cell.

Thank you for the clarification, this makes since.

5) The amount of PA26 or PA26PANc activation of the CP seems to be quite small, is it possible that the in vitro generated CPs are mostly in the open state to begin with, explaining why little activation is seen, and explaining why the distal end may appear open? A figure showing the amount of RP (all three) activation, on all CP's that are used in this manuscript would allow for a better assessment of the condition of the CP's that are being used.

The smaller activation in archaea compared to that of eukaryotic 20S is typical, and well documented in the literature, for example, Smith, et al. 2005 Molecular Cell and many other publications afterwards. As for the in vitro assembled 20S CP, the ratio of florescent intensities from CP with the activator and alone is comparable with the in vivo assembled 20S CP, as shown in Supplementary Figure 1k.

The authors cited, Smith et al. 2005 Molecular Cell, but this reference does not show fold stimulation by PA26 or by PA26PANc, which are shown in this manuscript and was the concern listed above. However, Yadon Yu et al. EBMO 2009 does shows 8-10 fold activation by these two complexes. The closed state of the 20S gate is sensitive to storage conditions, buffers, purification type etc. It appears to this reviewer that the 20S used here did not have stably closed gates, since RP's under saturating conditions produced only 2-3 fold activation. This also raises an alternative hypothesis that, which the authors data is also consistent with: perhaps the PA26panc has no effect on the distal 20S ring (since this gates are already in an open or disorder state), but perhaps PA26 allosterically closes the distal ring (while fully opening the proximal one). This model also explains the data that is observed (structural and biochemical), even though the conclusion is opposite to the authors presented here. More evidence is needed to rule out these alternative explanations, to make rigorous conclusions. In addition, I would like to point out the authors chose not to provide the requested verification experiments.

Indeed, Yadong Yu et al (EMBOJ, 2009, Figure 1D) showed that PA26 and PA26(E102A)PAN8 produced 6~7 fold activation. (Note that PA26(E102A)PAN8 is identical to the complex designated PA26PANc in this study.) In the current study, the activation of in vivo produced 20S by PA26PANc is ~5 fold, shown in the updated Supplementary 1k, which is comparable with what was reported in the EMBOJ 2009. Under the same condition, the activation of in vitro assembled 20S by the same activator is ~2.5 fold, roughly half that of in vivo produced 20S, as also shown in Supplementary 1k.

The initial comment was regarding the ~2.5 fold of the vitro complex. An 8 fold activation versus 2.5 is very different and suggests structural perturbations. [As requested by the Reviewer, we now included the structure of in vitro assembled 20S CP. No structure perturbation was observed.]

The reviewer raised the concern that the in vitro assembled 20S does not have a stable gate and further suggested an experiment to test this. In our previous response, we used the structural finding that the distal gate of PA26-αwtββαK66A complex is closed as evidence to support the conclusion that the gate of in vitro assembled 20S is fully intact and closed without activation. The Reviewer disagrees and raises another possibility, that the distal gate could be already open without activator but allosterically closed by binding of activator at the opposite α-ring. A direct test of this hypothesis would be to determine a structure of the asymmetric 20S αwtββαK66A proteasome without any activator. This is, however, not feasible by current state-of-the-art single particle cryo-EM technology. Without a bound activator, it is impossible to distinguish which α-ring in a particle image is αK66A or the wild type.

But one could tell if there was a difference in the state of the gate for the two different alpha rings… according to the authors. It wouldn't matter that they couldn't tell which was K66A. If the K66 mutation didn't effect gating then it should have identical alpha rings conformations. In addition the WT and K66A double ring mutant should show identical confirmations of the gate. [A structure of asymmetric 20S without activator would show that both gates are fully intact and closed, because it is the average of wild type and K66A mutant alpha rings. It thus cannot provide any evidence to prove or disapprove the gate conformation of the alpha ring with K66A mutation. Such an experiment would be meaningless and uninformative.]

The new hypothesis proposed by the Reviewer implies two concerns: in vitro assembled 20S proteasome does not have an intact gate, and/or K66A mutation causes the gate opening even without an activator. We address these two points separately with multiple experimental evidences:

First, we now show that the LFP hydrolysis of in vitro assembled 20S without activator (bar 4 of the updated Supplementary Figure 1k) is comparable (~12% lower) compared to the control, in vivo produced 20S without activator (bar 1). If the gate of in vitro assembled 20S is not stable, the LFP hydrolysis without activator should be significantly higher than the control.

However, it's not possible to determine proteasome concentration to such high accuracy between these completely different preparations so as to allow this conclusion. Functional differences between preparations is reasonable, but a determining that a 2 fold difference in activity is present is just not possible (or vice versa, that 2 fold difference in proteasome concentration could cause similar apparent activity). Typical protein determination techniques also account for contaminates, which typically very between preps. Also it's likely that mutation alter the percentage of active fraction for any enzyme. Thus making conclusions about small differences in basal activity between different preparations is just not possible. [There are two salient points here. Gate deletion (bar 3) and PA26PANc addition provide similar activities. In vivo and in vitro CP are both activated by PA26PANc. Both observations are consistent with the conclusion that the CPs used (except for the gate deletion mutant) have gates that are intact.]

Second, we now include in Supplementary Figure 2 a structure of in vitro assembled 20S without activator. This structure shows not only that the gate of in vitro assembled 20S is in closed conformation, the entire structure is identical with that of the in vivo produced 20S proteasome. Consequently, we conclude that the gate of in vitro assembled 20S is fully intact.

The fact that the in vitro assembled proteasome activates to a much lower extents suggests that the cryo EM technique here is unable to determine important structural differences that the biochemistry clearly shows. I think this highlights the limitation of cryo EM to distinguish the state of the gate in T20S complexes, which are highly dynamic, and exemplifies the primary issues that I am raising here. [We disagree. Gate conformation concerns whether a 20S CP is activated or not. It is not directly related to extent of biochemical activation. Indeed, it is not clear why the proteolytic activity of in vitro assembled 20S is lower, and this cannot be revealed at ~3Å resolution. However, our study concerns gate opening, which is addressable by cryo-EM.]

Third, as for the impact of K66A mutation on the gate configuration, it is indeed possible to determine a structure of the symmetric 20S with K66A in both □-rings. Unfortunately, we were unable to perform this experiment due to lab closure following the "shelter in place" order. With respect, we would like to point out the following evidence to prove that the K66A does not open the gate of 20S: Figure 5c of Foster et al (2005, Mol Cell) shows that, unlike the wild type, the peptidase activity of 20S proteasome with K66A cannot be activated by PA26. We quote the statement here: "The importance of αLys66 for binding was confirmed by demonstrating that αLys66Ala and αLys66Ser proteasome, both of which are correctly assembled as indicated by gel filtration and wild-type levels of activity against small fluorogenic peptides, do not bind PA26 in a velocity sedimentation assay (Figure 5B) and do not further stimulate proteasomal peptidase

activity (Figure 5C)." This suggests that K66A mutation alone does not alter the gate conformation from a closed to an open form.

The covid issues is unfortunate, however this does not address the original state alternative hypothesis for the preparations that the author is using.

We speculate that the lower activation, ~2.5 fold vs. ~5 fold, for in vitro assembled versus in vivo produced 20S CP, using the same activator, PA26PANc, reflects a lower proteolytic activity of in vitro assembled 20S CP. It is not unusual that an in vitro assembled complex is less active than that of an in vivo one. The absolute proteolytic activity of in vitro assembled 20S CP is not a concern in this study and does not change our conclusions.

It's not a major concern, agreed.

The newly included structure of in vitro assembled 20S proteasomes (Supplementary Figure 2) and updated Supplementary Figure 1k should, we believe, effectively address this concern of the Reviewer.

Not addressed for reasons mentioned above. [We disagree.]

6) The authors routinely use the argument that having a stoichiometric access of RP over CP indicates that the CP is saturated. This is incorrect. If the concentration of the CP or RP is below the Kd for this interaction than the molar excess is irrelevant to predicting saturation. Because of this it's impossible to predict what the fractional occupancy of the systems that are being used here, since no Kd's or affinity constants are determined. The authors argue that the EM images demonstrate saturation, but many particles can be seen that are not bound (sup fig 2), and in addition, most of the particles in these images are vertical, and thus their bound state cannot be determined. This justification for saturation is thus invalid. The authors should do dose response curves with each of their RP's on each of their CP variants to show where in the saturation curve these experiments are being done.

The Kd value of activator PA26 and PA26PANc binding to 20S proteasome are known from the literature. Kd of PA26PANc to 20S is 7pM, of PA26V230F to 20S is 3pM. In our experiments (Figure 2a and b, Figure 5g and Table 1) the concentration of 20S proteasomes used are in the nanomolar range, way above the Kd, thus ensuring that the CP is saturated. For all structural work, both negative stain and cryo-EM, the concentrations of final sample preparation are at nanomolar range. We thank the reviewer for pointing this out. We now revised the Methods section and included the concentration value in the Methods section.

While I understand that Stadtmueller et al. showed 3 and 7 pM affinities for these complexes (calculated via SPR), apparent affinities can vary greatly from prep to prep. In addition, while the authors argue there complexes are highly saturating (based on these reported affinities), the EM images simply don't agree with this statement as sup fig 2 shows many free 20S particles, which was mentioned above, but was ignored by the authors here. In addition, the above suggested experiments, to show that their conditions are actually saturating were ignored.

We thank the reviewer for pointing this out and apologize that we did not fully understand this comment during the previous round of revision. Indeed, it is likely that the Kd values of various activators binding to the 20S proteasome reported in previous publications by other investigators cannot be depended upon. These were determined by

surface plasmon resonance. These values may be inaccurate, or at least different from those relevant to our experiments, which were done with proteasome complexes in bulk aqueous solution, rather than tethered to a matrix, as in SPR experiments.

We have now performed additional experiments to measure the actual apparent binding affinities to 20S of all three activators used in our experiments, i.e., PA26, PA26PANc and PAN. These experiments were performed by measuring the dependence of peptidase activity on incubation with various concentrations of the three activators. From these experiments we calculated Kd-app values of activators for 20S. Using the Kd-app value, we can predict the fraction of α-rings that are bound with activator, the extent of 20S saturation by the individual regulatory complexes, by the formula Y (percentage) = [activator]/(Km +[activator]). In a fully independent method, we also used negative stain EM and 2D classification to determine the percentages of 20S particles that are doubly capped or singly capped. The overall percentage of capping estimated from negative stain EM is similar to what is estimated from Kd-app values described above.

As mentioned above using the Vmax values for the LFP data likely provides erroneous data. [Again, as mentioned before, we disagree.] **Also It's possible that double cap, single cap, and no cap complex absorb to the girds with different affinities, so counting via NS-EM is not rigorous (this was mentioned previously as well).** [This presumed artefact would have to apply differently for the PA26 and PA26PANc cases, as these give different results. These two RPs differ only in their last C-terminal amino acids, which are buried in the alpha ring and thus make unlikely an RP-dependent artefact of grid association. Virtually all key experiments are controlled by the comparison of PA26 versus PA26PANc.]

Using the Kd-app values inferred from the biochemical approach based on in vivo 20S, we estimated that ~83% of symmetric 20S proteasome are capped by PA26PANc (Figure 2a), ~72% are capped by PA26V230F (Figure 5g), and about 90% are capped by PAN (Figure 2c and d), respectively, under the experimental conditions used to collect our data. We believe that the percentage of particles capped is sufficiently high that the conclusions we drew from our experiments hold true. We now included this new information in the manuscript as Supplementary Figure 4.

We thank the reviewer for motivating us to perform these additional experiments. We believe including these quantitative estimations strengthens our manuscript significantly.

First these saturation estimates appear to be based on the saturation curves in supp fig 4, 2 of which are (as discussed above) completely inaccurate. [We disagree. The quality of the data provides estimations that fully address the question raised by the reviewer concerning the saturation by activator.] **In addition, 72-90% is certainly not saturated, especially when one is trying to assess the activity of and compare 50% bound (single caps) to 100% bound (double caps) proteasomes).** [Our comments above, we believe, offer sufficient rebuttal and do not require repetition.] **This is a concern, and these are only very loose estimates, and do not contain standard deviations. This is not strong evidence for the conclusions that are drawn. I appreciate the authors making these estimates. It's a shame they weren't done before the experiments, to allow for more conclusive biochemical results. The authors need to show a clear 2 fold change in activity, and a no change in activity to support their findings, but 72% saturation is just not going to allow this to happen. 99% bound should not be a problem to obtain biochemically.** [As addressed above, 99% saturation is not required for resolution of the questions under consideration.]

7) The experiment in Fig 5 show that presumed double cap PA26-CP particles have essential twice the activity of the single cap structures, but no data is shown indicating PA26 saturation (see #6 above). This doubling of activity is taken to indicate that 2 gates are being opened in the double cap, and 1 gate in the single cap (also see issues rasied in #2 here), but a more likely alternative explanation is: these experiments are done in the linear binding phase, such that the CP's allowing double cap formation provide 2Xs as many binding surfaces as do the CP's that only allow single cap formation. In this way twice the amount of binding (and activity) is observed. This reasonable alternative hypothesis is not ruled out in this study.

Similar to our response to point 6, the Kd of PA26 binding to 20S wild type alpha ring is 7pM. The concentration used in the experiments is mM. At this concentration, all CP wild type alpha rings are saturated with PA26. Again, we will include the Kd value in the manuscript to avoid potential misunderstanding.

The justification above (green) also applies here.

We hope the reply to the point 6 also addresses this comment.

The above reply only highlights this issues I have raised a 72% estimated saturation is certainly still in the linear phase of the hyperbolic curve, and it would be expected that doubling the amount of 20S binding sites (WT versus k66A mutant) would have a substantial effect on enhancing 20S-regulator binding, which could absolutely contribute to the observed 2 fold change with PA26. [Our comments above, we believe, offer sufficient rebuttal and do not require repetition.]

8) For cryo-EM analysis of the open channel state the authors use the pointing direction of the resolved alpha N-terminus to indicate if the gate is opened or closed (since most of the gating residues are unresolved. However, this approach does is not conclusive, since the presumed closed state (e.g. fig 5f) N-termini are not really pointing anywhere because the residues that point up (as in 5b) are simply NOT resolved in 5f. Absence of resolved structure cannot be assumed to exist in one state versus another.

Structurally, conformation of a closed gate in archaeal 20S proteasomes is always less well resolved than that of eukaryotic 20S proteasomes. This is because archaeal 20S proteasome itself has a D7 symmetry, except with a closed gate which does not follow the symmetry and do not form a specific conformation stabilized by hydrogen bound networks. Extensive structural studies in the past, including X-ray crystallography, cryo-EM and NMR, all reveal the same structural features of the gate: in a closed gate the first 10 ~ 11 N-terminal residues of alpha ring are not resolved (disordered), and the first resolved residues (typically Ile12) is pointing downward. When the gate is open, only the first 6 N-terminal residues are not resolved, and the Ala7 – Ala11 form a turn to point upwards. Related references include: Lowe et al. 1995, Science; Forster et al. 2003, EMBOJ; Smith et al 2005, Mol Cell; Rabl et al. 2008, Mol Cell, Huang, et al 2017, PNAS; Majumder et al. 2019, PNAS. A comparison of our structures with a known structure either with a closed or an open gate confirms the assigned gate conformation. We respectfully disagree with the reviewer: the assignment of gate conformation is clear without ambiguity.

While the author's arguments is well understood and reasonable, the absence of resolution is just very weak data to support the conclusion of a particular state. Other issues with obtaining high resolution in this area could also cause a loss of resolution, rather just a closed gate. If

other structural data was available to bolster this observation, the argument could be much stronger.

We appreciate that understanding of the Reviewer. We do believe that the structural data presented in this study are solid and the way we interpret our data is consistent with previous studies. Furthermore, they are supported by the biochemical data.

My opinion remains the same, 1) this is weak structural data, 2) that cryo EM may not be the right technique to make assessment of highly dynamic domains, and 3) that the biochemical data that are shown are weak as well or are incomplete. Combining two weak data sets, simply does not make these conclusions strong or rigorous, and are at best suggestive. [We disagree.]

9) Essential structural controls are missing in the Cryo-EM data. To demonstrate that the distal alpha-ring is opening when the proximal side is occupied by an RP, the authors must show the similar cryo-EM structure of the asymmetric (mutated) in vitro generated CPs in the unbound state. Only once these are obtained can a conclusive comparison be made to determine of allosteric gate opening is occurring. In addition, the comparison should be made via overlaying the electron densities (not atomic fits), to determine the real structural differences in the distal alpha ring. This is paramount.

We appreciate this comment. Determining structure of asymmetric 20S proteasome without any activator bound is impossible, as the alignment cannot distinguish which of the two alpha rings contains the K66A mutation. However, we do have the structure of asymmetric 20S with PA26 bound to the wild type alpha ring, and the gate of mutant alpha ring remains closed. This experiment does suggest that alpha ring with K66A mutation is closed, unless being allosterically opened. In addition, we can determine the structure of in vitro assembled symmetric 20S proteasome with the K66A mutation to show that its gate is closed, although we think such an experiment is unnecessary, because we already presented the structure of asymmetric 20S CP singly capped with PA26, where the alpha ring with K66A is closed.

I agree with the authors that the same 20S structure with the two different RPs PA26 & PA26panc are fairly conclusive, as one shows a distal open state and the other shows what appears to be a distal closed state (with caveats mentioned above). However, the authors also ignored our suggestion to show the overlaying electron densities (not atomic fits), of the two distal rings with these two different complexes. As mentioned before this reviewer believes this to be paramount for interpreting difference in these rings, which is the major conclusion of this manuscript. However, the authors ignored this request.

In our previous revision, we misunderstood the request of the Reviewer, and only presented the overlay of the atomic structure. In this revision, we included new panels in Supplementary Figure 6 the density of the distal gate of asymmetric 20S CP singly capped with three different activator, and compared it with the closed gate conformation of T20S without activator.

I appreciate the inclusion of this new analysis. Supp fig 6 shows no difference at all in the presumed open and closed rings, except for a small missing density in the closed state. A missing density is not rigorous evidence of a different state, in fact all other densities, look very similar. How can one expect a small terminus of a subunit to have different conformational state, when the rest of the subunit appears to be in the same conformation? If there were large conformal changes in the rest of the subunit, or

between subunits, there would be a cause for a change in the N-termini, but there doesn't appear to be. Also Figure 7K (which contains the overlay of the distal ring, which the authors argue is open, show almost no differences in the alpha-ring. However, the proximal ring sup fig 7j shows nice conformational changes, that could cause gate-opening. This also appears to be the same case for the PAN-20S structure changes shown in supp fig 8k. The distal ring overlay does not show conformational changes. In either complex, the rigorous evidence for an open state in the distal ring just isn't there.

[We disagree. As explained in the previous rebuttal/revision, and agreed by the Reviewer, the gate conformation determination is consistent with the criteria of all previous studies, which we cited in the previous round of rebuttals.]

10) It's not clear how the ratios of double, single and no cap CP's were calculated to indicate cooperative association. While the probability of double caps should be equal to the probability of single cap squared, it not clear that the probability calculation to determine the probability of free CPs is correct. More confusingly, the mathematical explanation in the text is different from that in the methods. A test case of the equation defined in the method: E.g. it suggested that the probability of alpha ring being bound is P = (2 □ E2 + E1)/2; were E1 and E2 are fraction of single and double caps; so if E1 was 0.5, and E2 was 0.25 than P = 0.5, but the fraction of alpha rings occupied are 0.75, not 0.5 in this case. The mathematical derivation should be clarified and better defined.

The calculation of the reviewer is incorrect. In the test case cited, assuming a total of 100 20S particles, 50 particles are singly capped, and 25 particles are doubly capped, and the remaining 25 are uncapped. Therefore, there are a total of 100 alpha rings that are capped: 50 alpha rings from single capped particles + 2 x 25 rings from doubly capped particles. There are 200 alpha rings in total, 2 alpha rings per particle. Therefore, the fraction of alpha rings occupied is 100/200 = 0.5, not 0.75.

Thank you for the clarification, this makes since.

Moreover, the data in Table 1 (if generated correctly) only indicate that a binomial distribution is not consistent with the single/double cap ratio's, but it does not indicate if this allostery is negative, positive, or to what extent (quantity) the allostery exists, which reduces the relevance of this analysis.

The data are strongly consistent with the conclusion that for a cap with the HbYX motif binding is positively cooperative. It is much less robustly suggestive that for the alternative cap without that motif binding may be negatively cooperative. We chose to discuss the more robust result.

So many factors can affect the distribution of these caps via negative stain EM. Do double caps absorb to the girds faster than single or no caps? Do no caps clump together, preventing section during counting? This data seems to be a good start, but should be verified via an independent method, such a one that can generate a hill coefficient from equilibrium binding in solution.

We also have the saturation problem here again, these conditions (according to the authors) are super saturating (~60X>Kd [140nm 20S at 3:1 RP:20S]), and if this is the case then by definition near 100% of the 20S should be bound to two RP's but their data in Table I show a large population of no cap structures. These data are not consistent with the reporting on affinity that is assumed here further justifying the prior concerns.

We appreciate that the saturation problem pointed out by the Reviewer. This is now fully addressed, as described in the response to point 6 above.

As discussed above, only 1 of the 3 data sets appear to be reliable to conclude near saturation was reached, and based on the authors estimates, the biochemical experiments were done at sub-saturating conditions. The detailed response is listed above. This concern still stands.

[Our comments above, we believe, offer sufficient rebuttal and do not require repetition.]

Can the authors show a Hill coefficient with statistical relevance? This would be far more convincing.

In designing the cooperativity experiments we reported, molar ratios of caps to 20S were chosen after preliminary experiments that would result in statistically meaningful populations of all three complexes: uncapped, singly capped and doubly capped. This is necessary so that the experimental data distribution can be compared with the theoretical distribution (nul hypothesis, no cooperativity of binding), using the appropriate statistical method, the chi square test. Calculating a Hill coefficient requires performing an impractically extensive analysis over a broad range of stoichiometric ratios of cap to 20S. The experiment as performed and reported is adequate to answer the question we posed. The conclusion that a cap with HbYX motif binds with positive cooperativity is robustly sustained at two different stoichiometric ratios of cap to 20S.

I disagree, for reasons as discussed immediately above.

This is also related to saturation/stoichiometry discussion, which is now fully addressed in point 6.

Not addressed for reason's mentioned above. The authors also do not simply provide the hill coef for regulator binding. The experiment was done, but no hill coef were calculated, and by just looking at the graphs, the data does not visually support a sigmoidal curve, suggesting that the PA26panc is not binding allosterically. This suggests that perhaps the negative stain data could be due to different grid absorption rates, for the single, double and no cap proteasomes, or other bias errors from particle picking.

[Determining the Hill coefficient is an interesting suggestion, and one that may be incorporated into our future studies. However, the biochemical assay required to do so measures response directly, and occupancy only indirectly. We chose instead a structural method that yields occupancy directly, scoring uncapped, singly capped and doubly capped complexes. In contrast, biochemical measurements and modeling require interpretation to yield occupancy and mechanistic insight. This is further complicated in the present case: unbiased fitting of data to models requires two parameters, Hill and a second that describes the relationship between occupancy and activity. According to our present analysis, this relationship is different for PA26 and PA26PANc. Given these limitations, a Hill coefficient is not the preferred way to estimate cooperativity of RP binding to CP. Regarding the absence of visual evidence supporting sigmoid binding, distinguishing hyperbolic (Hill = 1) versus sigmoid (Hill >1) requires clean data at low occupancy, the region of the saturation curve most informative for making that distinction. The experiments we performed were intended to measure degree of saturation; they therefore focused on [RP] approaching saturation. These data are thus not adequate for Hill coefficient

determination, and visual inspection is not informative in reaching a conclusion. The Hill coefficient is an indirect measure of cooperative ligand binding. The structural method we chose is in principle superior, as it gives direct information on the distribution of relevant ligand states.]

Minor problems:

1) Why wasn't biochemical data with PAN presented

With respect, it is not clear what biochemical data the reviewer means. We did show PAN degradation of GFPssrA by western blot in Figure 2.

The authors showed PA26, PA26panc with the peptide dose response, but did not also do this with PAN. It only makes since to do this with PAN, as the PAN-20S structure is also shown and used here to support their conclusions, and is the most cognate complex in this manuscript, and is the only biologically relevant complex.

Indeed, we did not perform the peptidase dose response experiment of PAN with in vitro assembled 20S proteasome. We thought it was unnecessary because, unlike PA26 and PA26PANc, the biological relevant substrate for 20S-PAN complex is GFPssrA. We did show GFPssrA degradation by in vitro assembled 20S (Figure 2c and d).

Now we also included comprehensive peptidase response of PAN with in vitro 20S (Supplementary Figure 4), with the purpose of determining the Kd-app. It also provides the data requested by the Reviewer.

The data shown in supp fig 4 for PAN is uninterpretable. It seems unfortunate the authors even bothered to show this data. Also, this data does not show cooperativity. The point of presenting the biochemical data for PAN was to show that the most physiologically relevant PAN-20S complex caused distal gate-opening due to across the 20S allostery. In other words: that double and single cap PAN complexes show the same activity. Which requires LFP usage at non-aggregating conditions (~5microM), and in conditions that produce single and double cap 20S that is saturated with PAN. However, even if this experiment was done properly under these boundaries, the authors would still have to demonstrate that 20S with two open channels are twice as active as those with 1 open channel, which the authors have not done, and is mathematically unlikely, for reasons discussed above. [Our comments above, we believe, offer sufficient rebuttal and do not require repetition.]

2) Why were mutation in the alpha subunit used that enhance rp binding when rp's readily bind the 20S anyway. Why was the V230F mutation used, PA26 already readily binds the CP with low nM affinity. These mutations are not well characterized, and it's not understood what mechanistic impact they would have. They do not seem justified.

With respect, we disagree. V230F mutation is well characterized and published in the literature. The mutation enhances the binding of PA26 to archaeal 20S proteasome and facilitates crystallization of PA26 with 20S proteasomes. Structural Models for Interactions between the 20S Proteasome and Its PAN/19S Activators. Stadtmueller BM, Ferrell K, Whitby FG, Heroux A, Robinson H, Myszka DG, Hill CP. J Biol Chem. 2010 Jan 1;285(1):13-7.

A single publication regarding affinity is not viewed as well characterized, and this still doesn't explain the necessity of its use. Could the data not be obtained with the WT complex?

The PA26 mutant is now characterized, as described in response to point 6 above.

Supp 4h has serious problems as discussed above, and is not reliable, and was not compared to WT PA26, so I still wouldn't call it characterized. The point of this concern was to justify the use of this mutant over WT, which was not justified. This point is minor.
[Again, our comments above, we believe, offer sufficient rebuttal and do not require repetition.]

3) PA26 is typically a strong activator of 20S gate-opening, but acceleration of substrate hydrolysis here seems to be very small. Is it possible that these CP's already exist in the open state by themselves?

Activation of in vitro assembled proteasome is in a similar range as reported in the literature as well as in our study. Our structural studies did show that the gate is closed and responsive to the activator binding.

I believe this to be incorrect, as addressed above.

This is now addressed in the response to point 2 above.

The counterpoint to this was presented above.

[Addressed above.]

4) Fig. 5 g indicates alpha-WT rings were used, but the table in "h" shows alphas with R28L. is this a typo or is different information shown in g and h. The text seems to treat "g" and "h" as corresponding data.

We thank the reviewer for pointing this out and have corrected it during revision.

5) Sup 7 does not indicate what the labels are for.

Particle being picked for calculation is labeled with a green dot. This is to show which particles are picked for calculation. We now added this information to the figure legend.

6) All supplemental figure legends are missing.

We apologize for this mistake.

7) Based on the 2D reconstructions and the written description in the main text, A is actually PA26PANc-αWTββαK66A and B is PA26PANc-αWTββαWT-PA26PANc.

We thank the reviewer for pointing this out.

8) Tables 2 and 3 are called out in the text but are missing.

We apologize for the mistake. Table 2 and 3 are now shown as Supplementary Table 3 and 4, which was somehow missing in the original submission. We now included them in the revision.

9) In vivo proteasome are referred to but not defined. It's assumed that they mean archaeal CP's expressed in e. coli, and not purified from archaea.

In vivo proteasome means full 20S proteasome expressed and purified from E. coli, instead of assembled in vitro by mixing different components. We have made this clear.

10) A table of the mutations discussed early in the results and their subsequent binding functions would clarify all of the mutations and provide a useful reference for future figures.

We thank the reviewer for this suggestion, and have added this information as the Supplementary Table 2.
